# Associated factors of myopia in a Portuguese sample of adolescents: Parental history and school-related lifestyle

**DOI:** 10.1371/journal.pone.0353283

**Published:** 2026-07-16

**Authors:** Amélia Fernandes Nunes, Mariana Cunha, Dário Ferreira, Miguel Castelo-Branco Sousa, Cristina Albuquerque Godinho

**Affiliations:** 1 Health Sciences Research Centre (CICS-UBI), University of Beira Interior, Covilhã, Portugal and Nova National School of Public Health, NOVA University Lisbon, Lisbon, Portugal; 2 Health Sciences Research Centre (CICS-UBI), University of Beira Interior, Covilhã, Portugal; 3 Center of Mathematics and Applications (CMA-UBI), University of Beira Interior, Covilhã, Portugal; 4 Academic Clinical Center of Beiras, and Health Sciences Research Centre (CICS-UBI), University of Beira Interior, Covilhã, Portugal; 5 Public Health Research Centre, Comprehensive Health Research Center, CHRC, NOVA University Lisbon, Lisbon, Portugal; University of Warmia, POLAND

## Abstract

**Purpose:**

This study aimed to investigate the interaction between parental myopia and school-related lifestyle patterns, and myopia in a sample of Portuguese adolescents. Based on previously reported data, we examined whether environmental and behavioural correlates traditionally associated with myopia, such as outdoor time, physical activity practice, screen time, and academic load, maintain their significance in a non-metropolitan European context.

**Methods:**

This analysis involved a sample of 874 adolescents (10−18 years). Myopia was measured using non-cycloplegic open-field autorefraction and defined as a spherical equivalent ≤ −0.50D in the worse eye. Potential factors correlated with myopia were collected through supervised questionnaires completed by students and parents. Data were analysed using univariate and multivariable logistic regression to identify independent associations adjusting for demographic and behavioural covariates.

**Results:**

The prevalence rate of myopia was 22.5%. The multivariable model identified family history of myopia as the strongest association (OR up to 3.75; CI:2.03–6.95; p < 0.001). Attending a higher school level (OR=2.20; CI:1.07–4.52; p = 0.032) and schools in urban locations (OR=1.59; CI:1.00–2.50; p = 0.048) showed a significant association. Regarding lifestyle, regular physical activity (≥ 2 times per week) was associated with odds of myopia (OR=0.47; CI:0.29–0.77; p = 0.003) even after adjustment for parental history of myopia. Although included in the fully adjusted model, factors such as smartphone use and breastfeeding did not reach statistical significance (p > 0.05).

**Conclusions:**

Despite the low population-density environment in this region, myopia prevalence was comparable to that reported in other European studies. The identified associations suggest that both parental history and school-related lifestyle factors should be considered when interpreting myopia patterns in adolescence. These findings may help inform school-based strategies aimed at promoting healthier visual and behavioural habits.

## 1. Introduction

The rising global prevalence of myopia has positioned it as a worldwide public health challenge, prompting growing apprehension among researchers, healthcare professionals, and society in general. This refractive error is major cause of vision impairment among young people, and its progression to high myopia increases the risk of vision-threatening complications in adulthood [1]. While the step rise in prevalence was most pronounced in East and Southeast Asia during the late 20th century, where it now appears to have plateaued, other regions, including parts of Europe, have shown more gradual but consistent increases [1,2]. Furthermore, it is noteworthy that the global prevalence of myopia in school-age children is high, affecting about one-third of this population [2].

Genetic factors have a significant influence on the development and progression of myopia [3,4]. However, the sharp rise in myopia rates among children, despite the stability of genetic factors, underscores the substantial impact of environmental and behavioural influences on the aetiology of the condition [3,5]. Myopia is considered a complex heritable ocular condition resulting from the interplay between genetic predisposition and environmental determinants, many of which remain the subject of ongoing investigation and debate [3,5–7]. Studies on environmental and lifestyle-related factors, such as excessive digital device use [8], sleep duration [9], and participation in sports [10], have produced less evident findings [11]; however, more consistent evidence links myopia to prolonged near work, reduced time outdoors, and the presence of myopic parents [5,7,12]. This highlights the need to move beyond simple exposure metrics to understand how lifestyle patterns interact with hereditary predisposition in specific environmental contexts.

Studying myopia in adolescence is particularly relevant, as recent evidence from the International Myopia Institute (IMI) indicates that myopia stabilization occurs later than previously estimated, with significant progression continuing into late adolescence in populations exposed to high educational demands [2,3]. This developmental window captures the cumulative impact of secondary education and changing lifestyle habits, making it a critical period for identifying the environmental and genetic factors that drive refractive errors.

Most research on myopia has been conducted in East Asian countries and in Singapore, where prevalence among children and teenagers can exceed 80% [13]. However, it raises questions about the generalizability of the findings to other parts of the world, particularly to regions with different cultural, environmental, and behavioural context. There is evidence that the presence of green areas around schools is associated with a lower risk of myopia in adolescents and a lower prevalence of myopia in schools [14]. Identifying and characterizing the factors associated with the myopia development is essential for implementing effective strategies to prevent condition [3].

Despite the predominant focus on Asian contexts, studies conducted in Portugal have also revealed a growing trend in myopia prevalence with age. Reported rates range from 6.5% in children aged 3–5 years [15], 12.7% among those aged 5–17 years [16], 21.5% in individuals aged 10–18 years [17], and 41.3% among young adults aged 18–24 years [18]. Studies on factors associated with myopia in Portuguese children are scarce. For instance, Sánchez-Tena et al. found a prevalence of 12.7% in a higly urbanized region, associated with family history and limited outdoor time [16].

In this context, the present study aims to investigate the associations between school-related context, lifestyle patterns, and myopia in adolescents. By incorporating parental history of myopia and behavioral metrics, to better identify potential lifestyle and genetic determinants associated with myopia. This study performs a secondary analysis of previous data from a larger sample [17], exploring the interaction between academic levels (2nd and 3rd cycles), outdoor time, screen time, and the geographical location of schools. Focusing on a regional sample from the central region of Portugal, we assess whether global myopia trends persist in settings with greater availability of natural light and green spaces, which are considered less ‘myopigenic’ than major metropolitan centers.

## 2. Methods

### 2.1. Participants

This secondary analysis of a previously described screening campaign [17] involved adolescents enrolled in the 2nd cycle (grades 5 and 6) and the 3rd cycle (grades 7, 8, and 9) of basic education in a city in the central region of Portugal. Students enrolled in grades 5–9 within the municipality were eligible to participate, provided that informed consent was obtained from a parent or legal guardian and verbal assent was given by the student on the day of the assessment. While previous regional reports focused on prevalence, the present study incorporates parental history of myopia and granular behavioral metrics (e.g., homework, physical activity, screen time) to identify potential lifestyle and genetic determinants associated with myopia in this adolescent sample.

The selection of these specific educational cycles, which typically correspond to ages 10–15, was intentional. Younger children (1st cycle, ages 6–9) were excluded to ensure the reliability of self-reported behavioral data and to minimize the potential for refractive errors associated with the high accommodative amplitude typical of early childhood, especially in the absence of cycloplegia. Although the standard age for these cycles is 10–15, the final sample included students up to 18 years old, reflecting the actual classroom composition which includes students with grade repetitions or delayed school entry. This age group represents a critical window of pubertal growth and increased academic demand, making it a priority for identifying school-based myopia risk factors. Exclusion criteria included: lack of parental questionnaire return; cognitive impairments interfering with questionnaire comprehension; and incomplete refractive measurements.

### 2.2. Instruments and equipment

Refractive error was measured without cycloplegia using an open-field autorefractor (PlusOptix, model A09), which measures refractive error automatically and binocularly at a distance of 1 meter. This minimizes the effects of proximal accommodation and generally produces less myopic results compared to closed-field devices [19]. Although non-cycloplegic autorefraction is known to potentially overestimate myopia, with recent evidence from Wilson et al. [20] indicating that this overestimate is, on average, less than 0.25D in children, this method has shown a strong correlation with cycloplegic retinoscopy and is considered reliable for myopia screening in children [20,21]. Furthermore, due to the school-based screening design, cycloplegic refraction was not feasible within the approved field protocol, as the administration of cycloplegic agents would have required on-site medical supervision and a clinical setup that was not available during the screening sessions.

For each student, three measurements were taken and the average of the spherical equivalent (SE) was calculated, defined as the spherical component plus half the cylindrical component. For students who wore refractive correction, the lens power was also measured with a digital lensometer (Nidek, model LM-500). If the PlusOptix device indicated that a student’s refractive error was beyond its measurable range, and if the child wore spectacles, we used the power of their usual lenses as an indicator of their usual refraction. For classification purposes, the spherical equivalent (SE) of the more ametropic eye was used. Myopia was defined as a spherical equivalent (SE) ≤ −0.50 diopters (D).

The potential factors correlated with myopia, were collected through a questionnaire. The instrument was developed based on an extensive literature review and refined by a multidisciplinary committee comprising eye care professionals, a physician, and a behavioral psychologist to ensure clinical relevance and minimize reporting bias. To assess clarity and cultural appropriateness, a pilot study was conducted with a representative class (data not included in the final analysis), leading to minor adjustments in wording. Although the questionnaire was not a previously validated standardized instrument, these steps were taken to enhance content relevance and comprehension. During the main data collection, the instruments were administered in a supervised classroom environment to minimize peer influence and social desirability bias. Information on perinatal and postnatal characteristics, as well as parental myopia history, was provided by the students’ parents, via printed forms distributed and collected by the class directors. Demographic, educational, and behavioral data were self-reported by the students. With the exception of age, questionnaire-derived variables were collected using predefined categorical response options. All response categories used in the analyses are presented in Table 2.

The study was conducted in the highest altitude region of mainland Portugal (average 83.5 inhabitants/Km^2^ [22]). School locations were classified based on *the Portuguese National Statistics Institute* (INE) criteria. Schools located in “Predominantly Urban Areas” were categorized as Urban, while those in “Moderately Urban Areas” were categorized as Rural/Semi-urban for analytical contrast within this regional context. No schools from “Predominantly Rural” were included in the study.

### 2.3. Data collection

The study included a standardized protocol for collecting refractive measurements, along with data on sociodemographic, educational, perinatal, and postnatal characteristics; parental history of myopia; and behavioral and environmental factors.

The data collected included demographic information such as age, sex, nationality, and geographic location of the school attended by the students. In the educational domain, data were recorded on the age at which the child began school, the grade level they attend, and the parents’ level of education. Perinatal and postnatal characteristics were also included, such as maternal gestational age, whether the child was born prematurely, and whether breastfeeding occurred. The history of myopia in the parents was also investigated. Regarding behaviors and lifestyle, the following were reported: average daily sleep time, average reading distance, frequency of physical activity, time spent on homework, daily time outdoors, and daily smartphone use. Additionally, information was obtained from teachers about special educational needs that could affect the ability to understand the questionnaire.

Ethical approval was obtained from the Ethics Committee of the National School of Public Health (Aproval CEENSP nº 29/2023), and authorization was granted by the Portuguese Ministry of Education. Data collection took place during the winter term of the 2024 school year. Written informed consent was obtained from the parents or legal guardians of all participating minors. In addition, verbal assent was obtained from the adolescents on the day of assessment. All participants were informed that participation was voluntary and they could withdraw at any time without penalty.

### 2.4. Statistical analysis

The data were analyzed using SPSS version 28 (IBM SPSS Statistics). Continuous variables were presented as mean ± standard deviation (SD), and categorical variables were reported as frequencies and percentages. The Mann-Whitney U test was used to compare means between groups, and the chi-square test (χ²) was applied to assess associations between categorical variables. Myopia prevalence was calculated for all sample characteristics.

To identify factors associated, only data from the more ametropic eye of each participant (the eye with the highest absolute spherical equivalent) were used for all statistical analyses. Myopia prevalence rates were calculated according to sex, education level, and other sample characteristics.

No imputation of missing data was performed. Descriptive and univariate analyses were conducted using all available observations for each variable; therefore, the effective sample size varied across analyses.

Variables were considered eligible for inclusion in the multivariable model based on a priori clinical and epidemiological relevance and/or a liberal screening threshold in univariable analysis (p < 0.1), in order to avoid excluding potentially important confounders at an early stage. Given the limited number of outcome events in the complete-case sample. The final model was kept parsimonious and fitted using binary logistic regression with the Enter method. Model parsimony was considered in light of the available number Events per Variable (EPV). Multicollinearity was assessed using variance inflation factors (VIF), with values <5 considered acceptable [23,24]. Odds ratios (ORs) with 95% confidence intervals (CIs) were calculated, and statistical significance was set at p ≤ 0.05 for all analyses.

## 3. Results

### 3.1. Sample description

**Fig 1:**
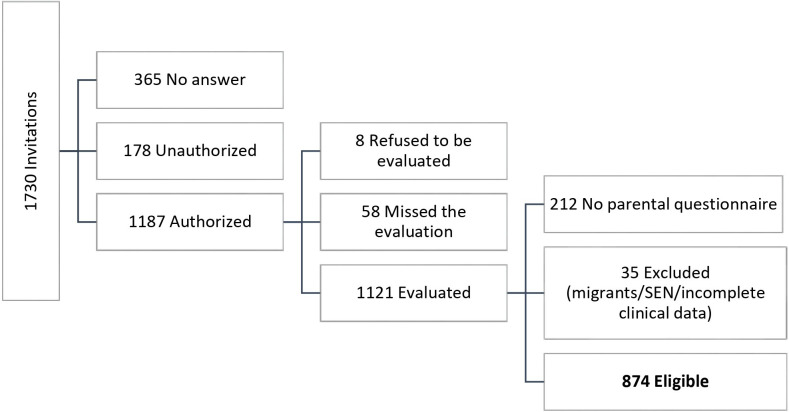
Flow diagram of participant recruitment and analytic sample selection.

A total of 1730 invitations were initially distributed, of which 1121 students were clinically evaluated. To ensure a robust multivariable analysis, a “complete-stages” approach was adopted, resulting in a final sample of 874 adolescentes (Fig 1). The main reason for exclusion was the absence of parental questionnaires (n = 212), wich were essential to assess hereditary predisposition. Although this selective approach impacts the final size, it significantly increases the internal validity of the reported behavioral and genetic associations reported.

The final sample comprise 874 students aged between 10 and 18 years (mean age: 12.6 ± 1.4 years). Of these, 52.3% were female, 40.2% were enrolled in the 3rd cycle of basic education, and 32.6% attended schools located in rural/semi-urban areas.

A detailed breakdown by age group is presented in Table 1. The majority of participants were between 10 and 14 years of age, with a gradual increase in myopia prevalence observed across age. Myopia affected 15,1.1% of 10–11 year-olds and rose to 26.7% among those aged 14 or older.

**Table 1:** Age distribution and myopia prevalence in the study sample.

Age (years)	N	With myopia – N (%)
10 - 11	304	47 (15.5)
12 - 13	379	99 (26.1)
14 and older	191	51 (26.7)
Total	874	197 (22.5)

**Table 2:** Descriptive characteristics and univariate associations with Myopia: demograthic, educational, perinatal/postnatal, and family history of myopia.

	Variable		N	With myopia	Without myopia	p-value	Crude OR (CI 95%)
	Total sample		874	197	677	--	--
	SE (diopters) [Mean ± SD]		−0.05 ± 1.85	−2.68 ± 1,84	0.72 ± 0.89	--	--
Demographic characteristics	Age (years)		12.6 ± 1.4	12.8 ± 1.3	12.5 ± 1.4	0.004**	**1.18 (1.05-1.32)**
	Sex	Girls Boys	417 457	93 (22.3%) 104(22.8%)	324 353	0.872	1.03(0.75-1.41)
	School location	Rural/Semi-urban Urban	285 589	52 (18.2%) 145(24.6%)	233 444	0.035*	**1.46(1.03-2.08)**
Educational characteristics	Age Starting School	≥ 72 months < 72 months	558 316	120(21.5%) 77 (24.4%)	438 239	0.331	1.18(0.85-1.63)
	Level of studies	2nd cycle 3rd cycle	351 523	59 (16.8%) 138(26.4%)	292 385	<0.001**	**1.78 (1.26-2.5)**
	Father’s Education	≤12 years >12 years	486 355	116(43.3%) 72 (38.3%)	370 283	0.218	0.81(0.58-1.13)
	Mother’s Education	≤12 years >12 years	363 497	82 (22.5%) 113(22.7%)	281 384	0.959	1.01(0.73-1.40)
Perinatal and postnatal characteristics	gestational age(mother)	<35 years ≥ 35 years	188 686	45 (23.9%) 152(22.2%)	143 534	0.605	0.91(0.62-1.32)
	delivery	Premature In term	83 752	21 (25.3%) 16622.1%)	62 586	0.503	1.20(0.71-2.02)
	Breast-feeding	No Breastfeeding < 6 months ≥6 months	94 288 443	18 (19.1%) 49 (17%) 118(26.6%)	76 239 325	0.007**	Ref 0.87(0.48-1.58) 1.53(0.88-2.67)
Family history of myopia	Neither parent is myopic Myopic father or mother Both parents myopic		279 316 93	34 (12.2%) 94 (29.7%) 30 (32.2%)	245 222 63	<0.001**	Ref **3.05(1.98-4.70)** **3.43(1.95-6.03)**

φThe total number for each variable may vary due to missing data (non-responses or ‘don’t know’ selections)

*significant at the 0.05 level; **significant at the 0.01 level

### 3.2. Factors associated with myopia

Some questionnaires on risk factors contained blank responses or had the “I don’t know” option selected. This occurred most frequently in the parental questionnaire, particularly in items related to family history of myopia, where approximately 20% of parents were unable to report whether they had myopia. For other variables, missing responses were occasional and randomly distributed.

Consequently, the effective sample size (N) varies across the factors analyzed. The effective sample size for each variable is provided in the second column of Tables 2 and 3. Odds ratio (OR) values for factors significantly associated with myopia are shown in bold.

**Table 3:** Descriptive characteristics and univariate sssociations with myopia: behavioural and environmental factors.

	Variable		N	With myopia	Without myopia	p-value	Crude OR (CI 95%)
	Total sample		874	197	677	--	--
	SE (diopters) [Mean ± SD]		−0.05 ± 1.85	−2.68 ± 1,84	0.72 ± 0.89	--	--
Behavioural and environmental characteristics	Sleep time	<8 hours ≥8 hours	342 521	87 (25.4%) 106(20.3%)	255 415	0.079	0.75(0.54-1.04)
	Reading distance	<30 cm ≥30 cm	204 655	55 (27.0%) 149(22.7%)	149 515	0.096	0.74(0.51-1.06)
	Practice physical activity	No practice 1 x week ≥2 x week	225 153 492	66 (29.3%) 39 (25.5%) 91 (18.5%)	159 114 401	0,003**	Ref 0.82(0.52-1.31) **0.55(0.38-0.79)**
	Homework Time	<1 hour day ≥1 hour day	518 352	109(21.0%) 88 (25.0%)	409 264	0.171	1.25(0.91-1.72)
	Outdoor time (week)	< 1 hour 1 to 2 hours ≥ 2 hours	105 323 445	25 (23.8%) 77 (23.8%) 94 (21.1%)	80 246 351	0.632	Ref 1.00(0.60-1.68) 0.86(0.52-1.42)
	Outdoor time (weekend)	< 1 hour 1 to 2 hours ≥ 2 hours	175 381 316	48 (27.4%) 87 (22.8%) 61 (19.3%)	127 294 255	0.117	Ref 0.78(0.52-1.18) **0.63(0.41-0.98)**
	Smartphone use (week)	<1 hour 1 to 2 hours 2 to 3 hours ≥ 3 hours	131 278 222 242	20 (15.3%) 58 (20.9%) 54 (24.3%) 65 (26.5%)	111 220 168 177	0.060	Ref 1.46(0.84-2.55) **1.78(1.01-3.14)** **2.04(1.17-3.55)**
	Smartphone use (weekend)	<1 hour 1 to 2 hours 2 to 3 hours ≥ 3 hours	104 219 229 320	13 (12.5%) 46 (21.0%) 62 (27.1%) 76 (23.8%)	91 173 167 244	0.026*	Ref 1.86(0.96-3.62) **2.60(1.36-4.98)** **2.18(1.16-4.12)**

φThe total number for each variable may vary due to missing data (non-responses or ‘don’t know’ selections).

*significant at the 0.05 level; **significant at the 0.01 level.

### 3.3. Multivariabe analysis

The results of the multivariable logistic regression, along with the variables included in the final model, are detailed in Table 4. The complete-case sample for the adjusted analysis comprised 651 participants with complete data on all included covariates. Among these, 147 had myopia, corresponding to an EPV ratio of 9.8 for 15 predictor parameters. Given the limited number of events, the model was kept parsimonious. All VIF values were <5, suggesting no major collinearity concerns, although age and school level showed moderate overlap.

**Table 4:** Multivariable logistic regression. (VIF – Variance Inflation Factor; aOR – Adjusted Odds Ratio; CI – Confidence Interval).

Included variables		VIF	p-value	aOR	CI 95%
Age (years)		3.339	0.742	1.04	0.82-1.33
Sex	Girls	1.070	0.373	0.83	0.55 - 1.25
	Boys				
School location	Rural/Semi-urban	1.021	0.048*	**1.59**	**1.00 - 2.50**
	Urban				
Level of studies	2nd cycle	3.283	0.032*	**2.20**	**1.07–4.52**
	3rd cycle				
Breast-feeding	Never	1.011	0,006 0.655 0.121	ref	
	< 6 months			0.85	0.42 - 1.73
	≥6 months			1.70	0.87 - 3.33
Family history of myopia	Neither parent	1.024	<0.001**	ref	
	Father or mother			**3.17**	**1.98–5.07**
	Both parents			**3.75**	**2.03–6.95**
Practice physical activity	No practice	1.088	0.003**	ref	
	1 x week			0.94	0.53 - 1.66
	≥2 x week			**0.47**	**0.29 - 0.77**
Smartphone use (week)	<1 hour	2.018	0.970	ref	
	1 to 2 hours			0.84	0.42 - 1.68
	2 to 3 hours			0.86	0.31 - 1.91
	≥ 3 hours			0.88	0.34 - 2.08
Smartphone use (weekend)	<1 hour	1.920	0.130	ref	
	1 to 2 hours			1.32	0.59 - 2.92
	2 to 3 hours			2.08	0.92 - 4.70
	≥ 3 hours			1.21	0.49 - 2.95

*significant at the 0.05 level; **significant at the 0.01 level

Although age was associated with myopia in univariable analysis, it did not remain significant after adjustment, likely because part of its effect was shared with school level, a closely related marker of educational exposure. Some behavioral variables discussed in the literature, such as weekend outdoor activity and homework time, did not meet the predefined threshold for entry into the multivariable model and were therefore not retained.

This analysis showed that several factors associated with myopia in the univariate analysis remained significantly associated after adjustment. Parental history of myopia remained the strongest correlate, with aORs of 3.17 (CI: 1.98–5.07) for one myopic parent and 3.75 (CI:2.03–6.95) for two myopic parents (p < 0.001). The association with school level was strengthened in the multivariable model, with students in the 3rd cycle showing over twofold higher odds of myopia (aOR = 2.20; CI: 1.07–4.52) and urban school location also maintained a significant association (aOR = 1.59; CI: 1.00–2.50). In terms of lifestyle, regular physical activity (≥ 2 times/week) was associated with lower odds of myopia (aOR = 0.47; CI: 0.29–0.77), corresponding to an approximate 53% reduction in odds. Other variables included in the model, such as breastfeeding duration and smartphone use (during the week and on weekends), did not remain statistically significant after adjustment (p > 0.05).

In a sensitivity analysis using a more conservative myopia definition based on spherical equivalent (SE ≤ −0.50 D) and visual acuity (VA ≥ 0.1 logMAR), the overall pattern of associations remained materially unchanged. Although school location was no longer statistically significant and weekend smartphone use reached statistical significance in the alternative model, the direction and overall magnitude of the associations were broadly similar to those observed in the primary analysis. Results from this sensitivity analysis are presented in S1 Table.

The multivariable logistic regression was based on a complete-case approach, including only participants with complete data for all covariates entered in the final model. Because family history of myopia was central to the study objective, it was retained in the adjusted model. A sensitivity analysis was additionally performed by re-running the multivariable model without family history of myopia. In this alternative model (n = 818), regular physical activity remained significant (aOR = 0.480; 95% CI: 0.319–0.720; p < 0.001), while associations with school cycle (aOR = 1.613; 95% CI: 0.881–2.953; p = 0.122) and urban school location (aOR = 1.295; 95% CI: 0.890–1.886; p = 0.176) lost statistical significance; furthermore, weekend smartphone use for 2–3 hours/day, compared with <1 hour/day, became statistically significant (aOR = 2.244; 95% CI: 1.056–4.767; p = 0.036), whereas the ≥ 3 hours/day category was not statistically significant. Full results are presented in Supplementary S2 Table.

## 4. Discussion

Our findings suggest that myopia in this adolescent sample was associated with both non-modifiable family history of myopia and school-related lifestyle patterns. While the observed prevalence of 22.5% (95% CI: 19.9%−25.4%) aligns with contemporary European data, the main contribution of this study lies in identifying parental myopia as the strongest correlate, together with an association between regular physical activity and lower odds of myopia. Even in a low-population-density region with high environmental availability of natural light, attendance at a higher school levels was associated with higher odds of myopia, which may reflect the influence of increasing academic demands. These findings suggest that school-related pressures may be relevant correlates if myopia even in environments considered less myopigenic.

In light of these findings, the prevalence of myopia observed in our study aligns with recent studies in Europe, where rates range from 10% in Sweden to 24.8% in Austria [25,26]. It is important to note that studies using cycloplegic refraction tend to report lower prevalence rates [25], whereas non-cycloplegic methods often result in higher estimates [17,26,27]. The 22.5% prevalence observed in our study falls within the range reported in the literature and suggests a relevant burden of the condition, even in a region with environmental characteristics, such as lower population density and more green spaces, considered favorable to protection against myopia [14]. Taken together, these findings place our results within the range of contemporary European studies, while suggesting that adolescents from non-metropolitan Portuguese settings may still exhibit myopia patterns comparable to those reported in more urbanized European populations.

Beyond hereditary predisposition, our univariate analysis confirmed that older age, attending urban schools, physical activity and prolonged weekend smartphone use, showed a significant correlation with, in line with other studies [8,27–31]. The lack of association with outdoor time may be due to the season during which data were collected, (i.e., in winter). In this study, smartphone use should be interpreted not as an isolated factor, but as an indicator of a broader digital and indoor lifestyle, possibly reflecting adolescents who spend less time in outdoor environments and more time in sedentary near-work activities [3]. Conversely, factors often debated in the literature, such as gestational age, breastfeeding, birth weight, or parental education level, [32–34] did not show significant associations in this specific sample. This lack of association suggests that contemporary behavioral habits and current educational pressures in adolescence may be more influential than early-life developmental factors for this specific age group. Among biological associations, parental myopia emerged as the strongest correlate (OR = 3.75; CI: 2.03–6.95, for both parents), in line with studies emphasizing a strong genetic contribution to myopia susceptibility [3–5,25]. Interestingly, while age was significant in the univariate stage, it lost significance when the school level was included in the final model. This suggests that in school-age populations, academic progression and its associated near-work demands (proxied by the school level) may be more influential than chronological age alone [11,28,35]. Our results showed an increase from 16.8% in the 2nd cycle to 26.4% in the 3rd cycle, suggesting that educational influences become more evident as academic demands escalate. This trend of increasing myopia with advancing educational stages is in line with findings from other international studies [35] and a Portuguese study conducted by Jorge et al., which documented a rise in myopia prevalence from 23.4% to 41.3% among university students between 2002 and 2014 [18]. Given that our study observed an increase in rates from the 2nd cycle to the 3rd cycle, it is plausible that myopia rates may continue to escalate in older student populations exposed to higher academic demands and prolonged near-work activities.

School location was also a key factor, supporting studies that report a greater prevalence in urban environments [30,31]. Notably, even in a region of relatively low population density, students in urban schools showed 1.6 times higher odds of myopia than those in rural/semi-urban settings (p = 0.048). This finding suggests thar the observed association with urban school location may reflect differences in local school-related environments and lifestyle habits, rather than population density alone.

Regarding behavioral factors, regular physical activity (≥ 2 times/week) was associated with lower rates of myopia (OR=0.47; CI: 0.29–0.77), corresponding to an approximate 53% reduction in odds. Although the literature often highlights time spent outdoors and smartphone use as relevant correlates of myopia [8,11,27,29], in our data outdoor activity did not meet the predefined threshold for inclusion in the multivariable model, whereas smartphone use was included butdid not maintain statistical significance after adjustment. This lack of significance, also observed in similar studies [16,28], may be attributed to the “complete-case” approach and the control of confounding effects. For exemple, the association observed for screen time may be partially explained by the higher academic demands of the 3rd cycle or attenuated by the strong influence of parental history in this specific sample. Similarly, breastfeeding duration was retained in the model but did not show an independent association after adjustment. These findings should therefore be interpreted cautiously and not as evidence of independent effects in this sample.

A strength of this study lies in the multivariable analysis, which was performed with an acceptable EPV ratio and low multicollinearity, supporting model stability and the assessment of independent associations between myopia and both modifiable and non-modifiable factors. Although the complete-case approach reduced the final analytic sample, all adjusted estimates were derived from the same set of participants. In addition, the sensitivity analysis (S1 Table) supported the consistency of the findings: when a more stringent definition of myopia was applied, the main associations remained materially unchanged, suggesting that the observed associations were stable across different thresholds for myopia classification.

Several limitations should be considered when interpreting our findings. 1)The primary limitation is the absence of cycloplegia. Myopia classification was based on open-field autorefractor measurements, which, although less prone to accommodative bias than closed-field devices, are not the gold standard for refractive assessment in this age group and may lead to an overestimation of absolute myopia prevalence [20]. Therefore, prevalence estimates from this study should not be interpreted as directely equivalente to those derived from cyclopegic protocols and may be slightly higher, particulary aroud spherical equivalente ≤ −0.50 D. Thus, the results should be interpreted with caution, given that the method used is appropriate for population screening and identification of risk patterns, but not for definitive clinical diagnosis [19]. We followed IMI guidelines (SE ≤ −0.50D) to aline our classification threshold with current epidemiological standards and facilitate cautious comparisons with other studies. At the same time, because the primary objective of this study was to examine associations within the same screened population, the internal pattern of associations remais informative, although the absolute prevalence estimate should be interpreted cautiously. 2) The final analytic sample (N = 651) was reduced by the complete-case approach, largely because of missing data in the parental questionnaire, particularly for family history of myopia. As these missing data may not have been completely random, some degree of selection bias cannot be excluded, and the magnitude of certain adjusted associations may have been affected. In addition, because participants were recruited through schools, in a single region of central Portugal, the sample should not be considered nationally representative. Although school setting/location was included as an adjustment variable, the limited number of schools precluded a more robust multilevel approach. While this may affect generalizability, the model maintained an acceptable Events Per Variable (EPV) ratio,supporting its statistical stability within this specific regional context. Therefore, the findings are most applicable to adolescents from similar non-metropolitan school settings and should be extrapolated cautiously to broad Portuguese adolescent population. 3) Our focus on smartphone use may not capture the full impact of all digital exposure. As different devices (e.g., computers tablets, or televisions) may envolve different viewing distances and visual demands, smartphone use in this study should be interpreted as a proxy for a broader indoor/digital lifestyle rather than an isolated factor. Future studies should explore how the different viewing distances and light intensities of various screens may relate to myopia in this age group. 4) The use of self-reported data for behavioral factors (outdoor time and physical activity) may have introduced recall or social desirability bias. In addition, the cross-sectional design precludes conclusions regarding temporal sequence or causality, and the observed associations should not be interpreted as evidence of directional effects. 5) Finally, residual confounding cannot be excluded, as some relevant variables were not directly measured in this study. Axial length, an important structural correlate of myopia, was not assessed because the screening protocol was designed for large-scale school-based refractive evaluation and did not support the use of higher-precision equipment such as ocular biometry. Likewise, outdoor exposure was assessed through self-reported time outdoors rather than objective light exposure measurements, and therefore may not fully capture actual ambient light exposure. Socioeconomic status was only partially represented by parental education level, while other potentially relevant indicators, such as household income, occupation, or material deprivation, were not available. These factors may have contributed to residual confounding and should be considered in the interpretation of the observed associations.

In conclusion, this study shows that myopia in our sample was associated with parental history and school-related lifestyle factors. Although the cross-sectional design of this study and the absence of cycloplegia require a cautious interpretation of the absolute prevalence estimate of myopia, the multivariable model suggests that parental history, school level, and physical activity are relevant correlates in this population. Parental history remains the strongest correlate, and the attendance at more advanced cycle of studies also independently associated with myopia. Regular physical activity, was associated with lower odds of myopia. These findings may help guide hypothesis generation and inform future school-based research.

## Supporting information

S1 Table Sensitivity analysis using conservative myopia criteria. Results of the multivariable logistic regression model using the alternative myopia definition (SE ≤ −0.50 D and VA ≥ 0.1 logMAR).
(DOCX)

S2 Table Sensitivity analysis excluding “Family History of Myopia” from the multivariable model. (DOCX)
