# Supplementary material for: Associated factors of myopia in a Portuguese sample of adolescents: Parental history and school-related lifestyle
Source: PLoS One. 2026 Jul 16;21(7):e0353283. doi: 10.1371/journal.pone.0353283 (PMC13375035; doi:10.1371/journal.pone.0353283)
Supplement: S1 Table — Results of the multivariable logistic regression model using the alternative myopia definition (SE ≤ −0.50 D and VA ≥ 0.1 logMAR). (DOCX) [file pone.0353283.s001.docx]

**S1 Table. Sensitivity analysis using conservative myopia criteria.** Results of the multivariable logistic regression model using the alternative myopia definition (SE ≤ -0.50 D and VA ≥ 0.1 logMAR).

| **Included variables** | | **p-value** | **aOR** | **CI 95%** |
| --- | --- | --- | --- | --- |
| Age (years) |  | 0.780 | 1.035 | 0.811 – 1.322 |
| Sex | Girls | 0.420 | 0.845 | 0.561 - 1.273 |
|  | Boys |  |  |  |
| School location | Rural/Semi-urban | 0.068 | 1.527 | 0.969 - 2.407 |
|  | Urban |  |  |  |
| Level of studies | 2nd cycle | **0.049*** | **2.058** | **1.003 - 4.222** |
|  | 3rd cycle |  |  |  |
| Breast-feeding | Never | 0.717  0.186 | ref |  |
|  | < 6 months |  | 0.877 | 0.432 - 1.780 |
|  | ≥6 months |  | 1.570 | 0.805 - 3.063 |
| Family history of myopia | Neither parent | **<0.001****  **<0.001**** | ref |  |
|  | Father or mother |  | **2.818** | **1,768 – 4.491** |
|  | Both parents |  | **3.589** | **1.945 – 6.621** |
| Practice physical activity | No practice | 0.719  **0.002**** | ref |  |
|  | 1 x week |  | 0.901 | 0.510 - 1.592 |
|  | ≥2 x week |  | **0.457** | **0.281 - 0.744** |
| Smart phone time (week) | <1 hour | 0.767  0.778  0.945 | ref |  |
|  | 1 to 2 hours |  | 0.900 | 0.447 - 1.811 |
|  | 2 to 3 hours |  | 0.892 | 0.402 - 1.980 |
|  | ≥ 3 hours |  | 1.031 | 0.436 - 2.433 |
| Smart phone time (weekend) | <1 hour | 0.344  **0,046***  0,666 | ref |  |
|  | 1 to 2 hours |  | 1.478 | 0.658 - 3.323 |
|  | 2 to 3 hours |  | **2.331** | **1.015 – 5.085** |
|  | ≥ 3 hours |  | 1.221 | 0.493 – 3.024 |

*significant at the 0.05 level; **significant at the 0.01 level
