# Supplementary material for: Associated factors of myopia in a Portuguese sample of adolescents: Parental history and school-related lifestyle
Source: PLoS One. 2026 Jul 16;21(7):e0353283. doi: 10.1371/journal.pone.0353283 (PMC13375035; doi:10.1371/journal.pone.0353283)
Supplement: S2 Table — (DOCX) [file pone.0353283.s002.docx]

**S2 Table. Sensitivity analysis excluding Family history of myopia from the multivariable model. (N=818)**

| **Included variables** | | **p-value** | **aOR** | **CI 95%** |
| --- | --- | --- | --- | --- |
| Age (years) |  | 0.795 | 1.03 | 0.84 – 1.26 |
| Sex | Girls | 0.426 | 0.87 | 0.61 - 1.23 |
|  | Boys |  |  |  |
| School location | Rural/Semi-urban | 0.176 | 1.30 | 0.89 - 1.89 |
|  | Urban |  |  |  |
| Level of studies | 2nd cycle | 0.122 | 1.61 | 0.88 – 2.95 |
|  | 3rd cycle |  |  |  |
| Breast-feeding | Never | 0.518  0.126 | ref |  |
|  | < 6 months |  | 0.82 | 0.44 - 1.51 |
|  | ≥6 months |  | 1.56 | 0.88 – 2.76 |
| Practice physical activity | No practice | 0.574  **>0.001**** | ref |  |
|  | 1 x week |  | 0.87 | 0.53 - 1.42 |
|  | ≥2 x week |  | **0.48** | **0.32 - 0.72** |
| Smartphone use (week) | <1 hour | 0.843  0.937  0.655 | ref |  |
|  | 1 to 2 hours |  | 0.94 | 0.50 - 1.77 |
|  | 2 to 3 hours |  | 0.97 | 0.48 - 1.98 |
|  | ≥ 3 hours |  | 1.19 | 0.56 - 2.51 |
| Smartphone use (weekend) | <1 hour | 0.266  **0,036***  0,279 | ref |  |
|  | 1 to 2 hours |  | 1.51 | 0.73 - 3.14 |
|  | 2 to 3 hours |  | **2.24** | **1.06 – 4.77** |
|  | ≥ 3 hours |  | 1.56 | 0.70 – 3.46 |

*significant at the 0.05 level; **significant at the 0.01 level
