# Supplementary material for: Associated factors of myopia in a Portuguese sample of adolescents: Parental history and school-related lifestyle
Source: PLoS One. 2026 Jul 16;21(7):e0353283. doi: 10.1371/journal.pone.0353283 (PMC13375035; doi:10.1371/journal.pone.0353283)
Supplement: Attachment — Submitted filename: ReplyReviewersvf.docx [file pone.0353283.s004.docx]

**Main Revisions Overview**

**We would like to thank the Academic Editor and the Reviewers for their thoughtful comments and constructive suggestions. These insights have been invaluable in improving the quality and clarity of our manuscript. The most significant changes include:**

**Title Update: To better reflect the cross-sectional nature of the study and the focus on the interplay between heredity and school-related lifestyle (as suggested by Reviewer 2), the title has been changed to: “Associated factors of myopia in a Portuguese sample of adolescents: Parental history and school-related lifestyle”**

**Data Availability: The dataset has been deposited in Zenodo (DOI:** <https://doi.org/10.5281/zenodo.19423653>**) and an institutional contact point has been provided.**

**Statistical Refinement: The multivariable analysis was refined using the "Enter" method, monitoring variance inflation factors (VIF) and "Events per Variable" (EPV) to ensure model stability**

**Sensitivity Analysis (Supplemental Material): In response to Reviewer 2, we have included a Supplemental Table featuring a more conservative classification system (Spherical Equivalent ≤ - 0.50D and Visual Acuity ≥ 0,1 logMAR). This additional analysis confirms the robustness of the associations identified in our primary model.**

**Discussion Overhaul:** Following the reviewers' suggestions, the Discussion section was entirely rewritten to be more concise, eliminate redundancies, and provide a more balanced interpretation of the results, focusing on the identified correlates and their practical implications.

**Response to Editor**

**Dear Editor,
Thank you for the opportunity to revise our manuscript, “Associated Factors of Myopia in Adolescents: The Interplay Between Heredity and School-Related Lifestyle in a Portuguese Sample” (formerly “Prevalence of Myopia and Associated Factors in Adolescents in a Central Region of Portugal”) (Manuscript ID: PONE-D-26-00450). We sincerely appreciate the constructive comments provided by the editorial team and the reviewers. We have carefully revised the manuscript and addressed the journal requirements and reviewer comments in the revised version.**

Adherence to Journal Requirements:

1. Style and Formatting: Ensure the manuscript meets PLOS ONE's style requirements, including file naming.

**Response:** We carefully reviewed the manuscript to ensure compliance with PLOS ONE style and formatting requirements, including manuscript organization, title page information, and file naming conventions. All relevant files have been revised accordingly.

2. Participant Consent Details: Provide additional details regarding participant consent (informed, type, minors).

**Response:** We have revised the Methods section (“Data Collection”) to provide additional details on participant consent. The manuscript now specifies that written informed consent was obtained from the parents or legal guardians of all participating minors, and that verbal assent was obtained from the adolescents on the day of assessment.

3. Data Availability and Institutional Contact: Revise the data statement to include a public repository and a non-author institutional contact point.

**Response:** We have revised the Data Availability Statement in accordance with the journal’s requirements. The anonymized dataset and accompanying data dictionary have been deposited in the Zenodo repository and are publicly accessible via DOI: <https://doi.org/10.5281/zenodo.19423653> . As requested, we have provided a non-author institutional contact point for data access and ethics inquiries: Contact the Data Protection Office (DPO) and the Cybersecurity Department of University of Beira Interior at prmi@ubi.pt.

4. Ethics Statement in the Methods Section: Include the full name of the IRB/Ethics Committee and details of consent in the "Methods" section.

**Response:** We have included the full Ethics Statement in the Methods section of the manuscript and clarified that written informed consent was obtained from the parents or legal guardians of all participating minors.

**Guide to the Revision**

Please note that page and line numbers in the following responses refer to the clean revised manuscript. All changes are also visible in the marked-up version with track changes.

**Response to Reviewer #1**

1. The introduction is weak and would benefit from a clearer rationale and concise review of relevant literature.

**Response:** We thank the Reviewer for this constructive suggestion. We have significantly revised the Introduction to provide a clearer rationale for the study. Specifically, we updated the second and final paragraphs, to integrate recent literature [11, 14] and emphasize the unique demographic context of our sample (lines 65-66; 79-84; 101-109).

2. Discussion of environmental factors should reference the recent review: DOI: 10.1186/s40101-024-00354-7.

**Response:** We agree with the Reviewer that this recent review is essential for contextualizing our results. We have now included the reference to Biswas et al., 2024 [11] in the introduction and Discussion section (lines 75, 322, 340). Furthermore, following the suggestions of all reviewers, the entire Discussion has been rewritten to place greater emphasis on the impact of environmental and behavioral factors while eliminating redundant information (lines 283-391).

3. The use of non-cycloplegic autorefraction in children (Lines 99–100) is a limitation, as Plusoptix may not be reliable across all refractive errors (Ref #19, OPO 2025).
**Response:** We agree that the use of non-cycloplegic refraction is a limitation. As noted in the recent review [19] and [20] although overestimation of myopia can occur, the difference is often small (generally <0.25 D), supporting the use of these devices in large-scale epidemiological screenings where cycloplegia is logistically and ethically unfeasible. We have updated both the Methods (lines 136-142) and the Limitations (lines 358-361) to reflect this point and to ensure a transparent interpretation of our findings.

4. Sample size is low, potentially limiting statistical power and generalizability.
**Response:** We understand the Reviewer’s concern. While the final analytic sample consisted of 651 participants (due to a rigorous complete-case approach), the multivariable model maintains a ratio of approximately 10 events per variable (EPV). This, combined with low multicollinearity (verified via VIF values), confirms the statistical stability and power of our analysis. Following the reviewers' suggestions, we have now used the "Enter" method for the multivariabel model and provided a detailed justification of our sample and model robustness in the Methods (lines 203 -213), Results (249 - 254), and Limitations (lines 365 - 371).

As requested by reviewer #2, we also clarified the novelty of this analysis and provided a participant flowchart (Figure 1), [more details in our response to Reviewer #2, Item 1].

5. Self-reported data from parents and children may be biased (especially lifestyle questions); the manuscript should clarify whether any validation or cross-checking was implemented.
**Response:** We agree that self-reported data can be subject to bias. To mitigate this, our data collection followed a standardized protocol: 1) the questionnaire was reviewed by a multidisciplinary committee of health professionals; 2) a pilot test was conducted with a separate class to refine the instrument; and 3) all questionnaires were completed in a classroom setting under the supervision of the research team to ensure consistency and clarify participant queries in real time. These details have been added to the Methods section (lines 152-162).

6. The criteria for classifying locations as protective against myopia or as urban/rural are unclear.

**Response:** We appreciate the Reviewer’s request for clarification. The geographical classification followed official Statistics Portugal (INE) criteria. In our study, "Rural/Semi-urban" refers to areas officially classified as "Moderately Urban," which in this low-population-density region retain traditional, low-density characteristics compared to major metropolitan centers. We gave more details in the Methods section (lines 163-167) and we have corrected the terminology throughout the manuscript to ensure consistency and clarity.

7. Numerical formatting is inconsistent; please standardize (preferably using decimals).

**Response:** We apologize for the oversight. We have carefully reviewed the manuscript and standardized the numerical formatting. All values are now presented using decimal points (e.g., 0.25 instead of 0,25) and a consistent number of decimal places for Odds Ratios (OR) and 95% Confidence Intervals (CI), as well as p-values, throughout the text and tables.

8. The rationale for selecting the 10–18 year age range is not provided.

**Response:** We appreciate the Reviewer’s request for clarification regarding the age range. The 10–18 year age range was selected to capture a critical period of myopia development characterized by increased academic demands and pubertal growth. We excluded younger children (ages 6–9) to ensure the reliability of self-reported data and to minimize the risk of refractive overestimation due to higher accommodative amplitude. The upper limit of 18 years accounts for grade repetitions within the sampled school cycles, ensuring a representative school-based cohort. We have added this rationale to the Introduction (lines 79–84) and Methods (lines 121–127).

9. Statistical analysis does not account for correlation between fellow eyes when using both eyes in logistic regression; appropriate methods should be applied.
**Response:** We entirely agree. To avoid violating the assumption of independence due to the high correlation between fellow eyes, our analysis used data from only one eye per participant. We selected the more ametropic eye (the one with the highest absolute spherical equivalent) for each subject, ensuring that each participant contributes only once to the model. This selection criterion has been clarified in the Abstract (line 36 - 37) and the Methods (lines 149 -151).

10. Overall: The study addresses an important topic, but methodological limitations and unclear reporting reduce the reliability and interpretability of the findings. Revision addressing these issues is recommended.

**Response:** We sincerely thank the Reviewer for the comprehensive and constructive feedback provided throughout this review process. We believe the manuscript's reliability and interpretability are now significantly enhanced.

**Response to Reviewer #2**

Dear Authors,
Thank you for the submission. The dataset is clearly valuable; however, because the underlying screening campaign has already been published, this manuscript must clearly differentiate itself and add substantial new value.
**Response:** Dear Reviewer,

We thank the Reviewer for the insightful evaluation of our work and for recognizing the value of our dataset. Following your suggestions, we have significantly shifted the focus from a prevalence-based report to a nuanced analysis of the interplay between heredity and school-related lifestyle factors. We have explicitly highlighted the novelty of this analysis in the Introduction and Discussion to ensure it provides substantial new value beyond previous publications. Our point-by-point responses are provided below.

1) Make the novelty explicit (required).
Please state explicitly (Abstract + Methods) that this is a secondary analysis/subsample of the previously published dataset, provide a participant flow (original screened N → analytic N), and add a short “What is new vs. prior publication” paragraph/table. Manuscripts based on the same database must substantially add and be transparent about overlap.
**Response:** We thank the Reviewer for the opportunity to clarify the distinct value of this work. We have explicitly updated the **Abstract** and **Methods** to state that this study is a secondary analysis of the database described in Nunes et al. (2024) [17].

While the previous publication focused on macro-epidemiology and correction coverage (Public Health focus), this manuscript provides a substantially new behavioral and genetic-environmental analysis. We have summarized the novelty in the table below:

We summarize the novelty in the table below:

| **Feature** | **Nunes et al. (2024)** | **Current Manuscript** |
| --- | --- | --- |
| **Scientific Focus** | Macro-epidemiology & Public Health | Parental myopia-Environment Interplay; Behavioral, Lifestyle & School-System Dynamics |
| **Variables** | Prevalence, Correction Coverage | Parental myopia, Homework duration, Reading distance, digital lifestyle patterns, Physical activity patterns |
| **Key Findings** | High percentage of uncorrected myopia | Physical activity as a significant independent protective factor against genetic risk |
| **Implication** | Need for better access to eye care | Need for school-based lifestyle interventions |

As requested, we clarified the novelty of this analysis in the Abstract (lines 31-34) and the final paragraph of the Introduction (lines 101-109). We have also included a participant flowchart (Figure 1) in the Results section to ensure full transparency regarding the analytic sample.

2) Shift the focus: this should be a “school system / digital exposure” paper, not another prevalence paper.
De-emphasize prevalence as a headline result and reframe around: differences between school cycles/grades and academic demands, weekend vs weekday digital device patterns, urban vs rural school context and how it structures behavior (outdoor time/physical activity), with clear, school-relevant implications (screening and prevention in schools). This is where the paper can genuinely stand out.

**Response:** We agree with the Reviewer’s perspective and have adjusted accordingly. The manuscript’s focus has been shifted from a prevalence-centered report to an association between myopia and school system factors, digital lifestyle, and parental myopia. Accordingly, the Discussion has been extensively restructured to emphasize the implications for school-based prevention and screening (lines 283–391).

In line with Reviewer #3’s suggestions, we also moderated the statements about prevalence and standardized the terminology throughout the manuscript (e.g., using ‘higher odds’ and ‘visual impairment’).

3) Screen-time interpretation: be precise.
You measure mainly smartphone/digital device time. Current evidence suggests device type matters and smartphone associations are not always the strongest signal compared with computers/TV; interpret as a proxy of indoor/digital lifestyle and discuss this limitation explicitly.
**Response:** We appreciate this insightful point regarding the differentiation of digital devices. Following your suggestion, we have updated the Discussion section (lines 308–310) to interpret smartphone and digital device use as a proxy for a broader "digital and indoor lifestyle." We explicitly discuss that this behavior likely reflects a displacement of time spent in protective outdoor environments. Furthermore, we added a paragraph to the Limitations (lines 372–376) acknowledging that while our study highlights digital patterns, we did not differentiate between all device types (e.g., smartphone vs. TV), which may yield different associative strengths as suggested by current evidence.

4) Fix technical credibility issues (before any interpretation).
Correct the implausible/inconsistent counts in the tables and ensure p-values/OR/CI are internally consistent. Then strengthen modelling (avoid/justify stepwise selection; account for school clustering; sensitivity analysis given non‑cycloplegic refraction threshold)

**Response:** We thank the Reviewer for this rigorous technical critique. We have addressed these concerns as a priority before proceeding with any interpretation of the results:

1. Data Consistency: All tables were meticulously audited. We corrected any inconsistencies between counts, p-values, Odds Ratios (ORs), and 95% Confidence Intervals (CIs) to ensure total internal consistency.
2. Modeling Refinement: As suggested, we replaced the stepwise selection with the "Enter" method for the multivariable logistic regression. We also verified that the final model maintains an Events Per Variable (EPV) ratio > 10, ensuring statistical stability.
3. Multicollinearity & Age vs. School Cycle: We assessed collinearity using VIF values. Due to the high correlation between age and school cycle (VIF > 3), we performed a sensitivity analysis excluding age. In this refined model (VIF = 1.19), school cycle remained significantly associated with myopia (OR = 2.10; 95% CI: 1.32–3.34; p = 0.002), suggesting that academic progression is a more robust correlate than chronological age in this sample.
4. School Clustering: While we acknowledge the potential for within-school correlation, the limited number of schools included precluded a robust formal multilevel approach. To account for this, school location was retained as an adjustment variable in the model, and we have acknowledged the possibility of residual clustering as a limitation.
5. Sensitivity Analysis (Non-cycloplegic Refraction): To address the refraction threshold concern, we conducted a sensitivity analysis using a more conservative myopia definition (Spherical Equivalent combined with Visual Acuity). The results, now provided as Supplemental Material (S1 Table), show that the overall pattern and magnitude of associations remain materially unchanged, supporting the robustness of our primary findings.

These updates are reflected in the Methods (lines 204–213), Results (lines 242–280), and Limitations sections (lines 365-371).

**Response to Reviewer #3**

We thank Reviewer #3 for the rigorous and detailed evaluation of our manuscript. The comments provided were fundamental in refining our scientific terminology, simplifying the discussion, and ensuring the technical soundness of our statistical modeling. Our point-by-point response is presented below.

a) Major Points
   1 Cycloplegia / refractive assessment: Refractive error was measured without cycloplegia. Although the use of an open-field autorefractor may reduce accommodative effects, the absence of cycloplegia remains an important limitation in this age group and should be clearly acknowledged due to the potential overestimation of myopia prevalence.
   **Response:** We agree that the absence of cycloplegia is a significant methodological limitation. While open-field autorefraction was used to minimize instrumental accommodation during this large-scale screening, we have now explicitly acknowledged this limitation in the Methods (lines 133-142), and Discussion (lines 358-365). To further address this, we integrated recent evidence [19, 20] regarding the expected bias and, as suggested by Reviewer #2, shifted the manuscript's focus from a prevalence-based report to an analysis of behavioral and environmental associations. We have also moderated all prevalence-related claims throughout the text.

2 Interpretation of prevalence: The reported prevalence of 22.5% falls within the range described in other European studies. Statements describing this prevalence as “significant” or implying a strong public health burden should be moderated.
**Response:** We agree with the Reviewer’s assessment. We have carefully revised the manuscript to ensure a more cautious and neutral interpretation of our findings. Terms such as "significant" or "alarming" have been replaced with descriptive language that contextualizes our results (22.5%) within the ranges of contemporary European data. This moderation is reflected throughout the Abstract, Results, and Discussion sections.

3 Interpretation of associated factors: Some associations should be interpreted with greater caution. For example, the association with classroom seating position may reflect reverse causation (students with myopia may preferentially sit closer to the board) rather than a true risk factor.

**Response:** We are grateful to the Reviewer for this insightful observation regarding reverse causation. We fully agree that classroom seating position is a confounded variable, as students with pre-existing myopia likely sit closer to the board to compensate for their visual impairment. To maintain the scientific integrity of our findings and avoid misleading interpretations, we have removed this variable from all statistical analyses. All tables, results, and discussion points have been updated accordingly.

4 Multivariate model interpretation: The reporting of the multivariate model could be clarified. Variables such as breastfeeding and smartphone use appear in the final model despite not reaching statistical significance. The manuscript should clearly distinguish between variables retained in the model and those with statistically significant associations.
**Response:** Following the reviewers' suggestions, we replaced the stepwise selection with the "Enter" method to provide a more robust and transparent multivariable model. As per standard epidemiological practice, variables that showed a p-value < 0.25 in the univariate analysis (such as breastfeeding and smartphone use) were retained in the final model to ensure full adjustment, regardless of their final statistical significance. This approach prevents the omission of potential confounders and provides a clearer picture of independent associations. We have clarified the distinction between retained variables and statistically significant associations in the Methods section (lines 207–213) and updated the Results accordingly in Table 3.

5 Length of the discussion and conclusion: The discussion is relatively long and could be streamlined. Several sections repeat results already presented in the tables. The conclusion could also be shortened and focus more directly on the main findings rather than broader public health implications that are not directly supported by the study design.
**Response:** We appreciate the guidance to improve the manuscript's conciseness and flow. Both the Discussion and Conclusion have been significantly streamlined. We removed redundant descriptions of the results and refocused the narrative on our primary findings: the role of hereditary predisposition, academic progression, and physical activity. Furthermore, we revised the Conclusion to align more strictly with the study’s scope, focusing on the observed data rather than broad public health implications.

b) Minor / Specific Points
1 Abstract
• The conclusion is somewhat long and could be shortened to better reflect the key findings.
• The absence of cycloplegic refraction should be mentioned as an important limitation.
• Associations such as classroom seating position should be interpreted cautiously.
**Response:** We revised the Abstract accordingly. The Conclusion was simplified to highlight our primary findings (heredity, school cycle, and physical activity). The absence of cycloplegia is now explicitly mentioned as a significant limitation. As per our previous response, the variable regarding classroom seating position was removed from the analysis to avoid bias from reverse causation, and is no longer mentioned in the Abstract.

2 Introduction
• The introduction could be streamlined to improve clarity and avoid overly strong statements.
• For example, describing myopia as the “leading cause of correctable vision loss among young people” seems overly strong; wording such as vision impairment would be more appropriate.
• The specific knowledge gap addressed by the study should be stated more clearly.
• Please clarify the rationale for focusing specifically on adolescents aged 10–18 years.
• The phrase “estimate the proportion of adolescents with myopia” could be replaced with “estimate the prevalence of myopia.”

**Response:** We revised the Introduction to ensure a more neutral and precise tone. Specifically, we updated the terminology from “vision loss” to “visual impairment” and replaced ‘proportion’ with ‘prevalence’, as suggested. We added the rationale for focusing on adolescence, highlighting it as a critical period for academic transition (lines 79-84).

3 Methods
• The contextual description of the study region (e.g., altitude, population density) could be shortened, as these variables are not directly analyzed in the study.
**Response:** The contextual description of the study region was simplified. We removed geographical data such as altitude, keeping only essential information about the school environment and geographical characteristics to provide the necessary context for the participants' environment.

4 Results
• Parts of the narrative repeat information already shown in the tables (particularly Table 2). The text could focus more on the key findings.
• Some wording could be simplified (e.g., replace “greater probability of myopia” with “higher odds of myopia”).
• The terminology used for breastfeeding categories should be revised. Expressions such as “Didn’t do it” are not appropriate for a scientific manuscript and could be replaced with “No breastfeeding.”
**Response:** We significantly revised the Results section to improve clarity and accuracy. We simplified the wording by removing redundant descriptions of data already presented in the tables, focusing on the main findings. We also standardized the terminology throughout the text and tables, replacing “higher probability” with “higher odds”. In addition, the breastfeeding categories were corrected; informal expressions such as “Did not do this” were replaced with “No breastfeeding” to ensure a professional scientific tone.

5 Discussion
• Some interpretations appear slightly overstated and could be moderated.
• Several paragraphs could be shortened, particularly where findings are repeatedly compared with the literature.

**Response:** We significantly revised the Discussion section to ensure a more concise and balanced interpretation of our results, following several suggestions from reviewers. Several paragraphs were simplified, reducing repetitive comparisons with existing literature and focusing on the main associations identified in our sample. We also moderated the tone of our interpretations, ensuring that conclusions about the associations of myopia with behavioral and school system characteristics are clear, avoiding overestimating the impact on public health.
