# Supplementary material for: Associated factors of myopia in a Portuguese sample of adolescents: Parental history and school-related lifestyle
Source: PLoS One. 2026 Jul 16;21(7):e0353283. doi: 10.1371/journal.pone.0353283 (PMC13375035; doi:10.1371/journal.pone.0353283)
Supplement: Attachment — Submitted filename: Response to Reviewers_2nd.doc [file pone.0353283.s005.doc]

**PONE-D-26-00450R1**

**Associated factors of myopia in a Portuguese sample of adolescents: Parental history and school-related lifestyle**

**PLOS One**

**Reviewers' comments and Response:

6. Review Comments to the Author**

**Reviewer #4: Thank you for your invitation to review manuscript entitled" Title: Prevalence of Myopia and Associated Factors in Adolescents in a Central Region of Portugal**

**1. Use of Non-Cycloplegic Refraction (Key Methodological Limitation)
The study measures myopia without cycloplegia, which is known to overestimate myopia prevalence, particularly in children and adolescents due to accommodation bias.
• Strengthen justification for this choice in the Methods.
• Expand discussion on potential overestimation magnitude.
• Clearly state how this limitation affects comparability with other studies.**

**Response:**

We thank the reviewer for this important comment. We agree that the absence of cycloplegia is a methodological limitation in refractive assessment in children and adolescents, as it may lead to an overestimation of myopia prevalence.

To address this point, we made two focused clarifications in the manuscript. First, in the Methods section, in addition to justifying the use of open-field autorefraction without cycloplegia, we now state that cycloplegic refraction was not feasible within the approved school-based field protocol because there was no on-site medical supervision for the administration of cycloplegic agents. Second, in the Limitations section, we clarify that prevalence estimates derived from our non-cycloplegic protocol should not be considered directly equivalent to those obtained under cycloplegic refraction and may be slightly higher, particularly around threshold-based classifications such as spherical equivalent ≤ -0.50 D. At the same time, because the main objective of the present study was to examine associations with family history and lifestyle factors within the same screened population, the internal pattern of associations remains informative, although the absolute prevalence estimate should be interpreted cautiously.

These clarifications have been incorporated into the Methods and Discussion sections.

Methods: (xxx)

*“Furthermore, due to the school-based screening design, cycloplegic refraction was not feasible within the approved field protocol, as the administration of cycloplegic agents would have required on-site medical supervision and a clinical setup that was not available during the screening sessions.”*

Discussion/Limitations (xxx)

*“The primary limitation of this study is the absence of cycloplegia. Myopia classification was based on open-field autorefractor measurements, which, although less prone to accommodative bias than closed-field devices, are not the gold standard for refractive assessment in this age group and may lead to an overestimation of absolute myopia prevalence [20]. Therefore, prevalence estimates from this study should not be interpreted as directly equivalent to those derived from cycloplegic protocols and may be slightly higher, particularly around threshold-based classifications such as spherical equivalent ≤ -0.50 D. Thus, the results should be interpreted with caution, given that the method used is appropriate for population screening and for identifying risk patterns, but not for definitive clinical diagnosis [19]. We followed IMI guidelines (SE ≤ -0.50D) to align our classification threshold with current epidemiological standards and facilitate cautious comparisons with other studies. At the same time, because the primary objective of this study was to examine associations within the same screened population, the internal pattern of associations remains informative, although the absolute prevalence estimate should be interpreted cautiously.”*

**2. Cross-Sectional Design and Causality
The cross-sectional design limits causal inference, yet some interpretations suggest directional relationships (e.g., smartphone use → myopia).
• Rephrase conclusions to emphasize association, not causation.
• Avoid causal language such as “increases risk” or “protective effect” without caution.**

**Response:**
We thank the reviewer for this important comment. We agree that the cross-sectional design of the study does not allow causal inference or temporal interpretation of the observed relationships. In response, we carefully revised the manuscript to ensure that all interpretations are framed in terms of association rather than causation.

These changes were incorporated throughout the manuscript to ensure that the findings are interpreted strictly as associations within the studied population.

Furthermore, we reformulated the statements in the Abstract, Discussion, and Conclusion to avoid directional or causal terms.

Abstract (results):

*“Regarding lifestyle, regular physical activity (≥ 2 times per week) was associated with odds of myopia (OR=0.471, p=0.003) even after adjustment for parental history of myopia.”*

Abstract (conclusion):

*“Despite the low population-density environment in this region, myopia prevalence was comparable to that reported in other European studies. The identified associations suggest that both, parental history and school related lifestyle factors, should be considered when interpreting myopia patterns in adolescence. These findings may help inform school-based strategies aimed at promoting healthier visual and behavioural habits.”*

Discussion (1^st^ paragraph):

*“Our results suggest that myopia in this adolescent sample was associated with both non-modifiable familial factors and school-related lifestyle patterns. Although the observed prevalence of 22.5% (95% CI: 19.9%–25.4%) is in line with contemporary European data, the main contribution of this study lies in identifying parental myopia as the strongest correlate, together with an association between regular physical activity and lower odds of myopia. Even in a low-population-density region with high availability of natural light, attendance at a higher school cycle was associated with higher odds of myopia, which may reflect the influence of increasing academic demands. These findings suggest that school-related pressures may be relevant correlates of myopia even in environments traditionally considered less myopigenic.”*

Conclusion:

*“In conclusion, this study shows that myopia in our sample was associated with parental history and school-related lifestyle factors. Although the cross-sectional design of this study and the absence of cycloplegia require a cautious interpretation of the absolute prevalence estimate of myopia, the multivariable model suggests that parental history, school cycle, and physical activity are relevant correlates in this population. Parental history remained the strongest correlate, and attendance at a higher school cycle was also independently associated with myopia. Regular physical activity was associated with lower odds of myopia. These findings may help guide hypothesis generation and inform future school-based research.”*

**3. Handling of Missing Data
Approximately 20% missing data for parental myopia is substantial.
• Explain how missing data were handled (e.g., exclusion, imputation).
• Include sensitivity analysis if possible.
• Discuss potential bias introduced by missing parental data.**

**Response:**
We agree that the proportion of missing data for parental myopia is substantial. In our dataset, missing data occurred primarily in the parents' questionnaire, particularly in the item on parental myopia, where some parents selected "Don't know" or left the item unanswered.

We now clarify in the manuscript that no imputation was performed. Descriptive and univariate analyses used all available observations, whereas the multivariable model was based on a complete-case approach. This model (n=651) retained parental myopia because it represents a variable that showed a strong association in the univariate analysis, and also because this association is well-documented in the literature.

As a sensitivity analysis, we reanalysed the multivariable model excluding parental myopia. In this alternative model (n=818), regular physical activity remained significant, while associations with school cycle and urban school location lost statistical significance; furthermore, smartphone use for more than 2 hours on weekends became significant. We now recognize, more explicitly, that the absence of data on parental myopia may not have been entirely random and may have influenced the magnitude and statistical significance of some environmental and behavioural associations. These clarifications have been incorporated into the Methods, Results, and Limitations sections, and the complete sensitivity analysis is shown in Supplementary Table S2.

Methods:

*“No imputation of missing data was performed. Descriptive and univariate analyses were conducted using all available observations for each variable; therefore, the effective sample size varied across analyses.”*

Results:

*“The multivariable logistic regression was based on a complete-case approach, including only participants with complete data for all covariates entered in the final model. Because family history of myopia was central to the study objective, it was retained in the adjusted model. A sensitivity analysis was additionally performed by re-running the multivariable model without family history of myopia. In this alternative model (n = 818), regular physical activity remained significant (aOR = 0.480; 95% CI: 0.319–0.720; p < 0.001), while associations with school cycle (aOR = 1.613; 95% CI: 0.881–2.953; p = 0.122) and urban school location (aOR = 1.295; 95% CI: 0.890–1.886; p = 0.176) lost statistical significance; furthermore, weekend smartphone use for 2–3 hours/day, compared with <1 hour/day, became statistically significant (aOR = 2.244; 95% CI: 1.056–4.767; p = 0.036), whereas the ≥3 hours/day category was not statistically significant. Full results are presented in Supplementary S2 Table.”*

***S2 Table. Sensitivity analysis excluding Family history of myopia from the multivariable model.***

Limitations:

*“The final analytic sample (N = 651) was reduced by the complete-case approach, largely because of missing data in the parental questionnaire, particularly for family history of myopia. As these missing data may not have been completely random, some degree of selection bias cannot be excluded, and the magnitude of certain adjusted associations may have been affected.”*

**4. Variable Selection in Multivariate Model
The manuscript does not clearly describe:
• Criteria for including variables in the multivariate model
• Whether collinearity was assessed
Amended
• Specify selection criteria (e.g., p < 0.20 in univariate analysis).
• Report multicollinearity diagnostics (e.g., VIF).
• Clarify why some variables (e.g., age, outdoor time) lost significance.**

**Response:** **We thank the reviewer for this important comment. We agree that the multivariable model-building strategy required clearer explanation. In the revised manuscript, we now specify that variables were considered eligible for the multivariable model based on a priori clinical and epidemiological relevance and/or a screening threshold of p < 0.10 in univariate analysis. This approach was adopted to avoid excluding potentially relevant covariates too early, while still preserving model parsimony given the limited number of outcome events in the complete-case sample.**

**Multicollinearity was assessed using variance inflation factors (VIF), and these diagnostics are now more explicitly reported and interpreted. In particular, age and school cycle showed moderate overlap, which likely explains why age lost statistical significance after adjustment.**

**We also clarified the handling of behavioural variables. Outdoor activity and homework time did not meet the predefined threshold for entry into the multivariable model and were therefore not retained. This point is now made explicit in the manuscript to distinguish variables that were excluded before model fitting from those that were included but did not remain statistically significant after adjustment.**

**These clarifications have now been incorporated into the Methods and Results section.**

Methods (statistical analysis)

*“To identify factors associated, only data from the more ametropic eye of each participant (the eye with the highest absolute spherical equivalent) were used for all statistical analyses. Myopia prevalence rates were calculated according to sex, education level, and other sample characteristics.*

*No imputation of missing data was performed. Descriptive and univariate analyses were conducted using all available observations for each variable; therefore, the effective sample size varied across analyses.*

*Variables were considered eligible for inclusion in the multivariable model based on a priori clinical and epidemiological relevance and/or a liberal screening threshold in univariate analysis (p<0.1), in order to avoid excluding potentially important confounders at an early stage, given the limited number of outcome events in the complete-case sample. The final model was kept parsimonious and fitted using binary logistic regression with the Enter method. Model parsimony was considered in light of the available number Events per Variable (EPV). Multicollinearity was assessed using variance inflation factors (VIF), with values <5 considered acceptable [23,24]. Odds ratios (ORs) with 95% confidence intervals (CIs) were calculated, and statistical significance was set at p≤0.05 for all analyses.”*

Results: Multivariable analysis:

*“Although age was associated with myopia in univariate analysis, it did not remain significant after adjustment, likely because part of its effect was shared with school cycle, a closely related marker of educational exposure. Some behavioural variables discussed in the literature, such as weekend outdoor activity and homework time, did not meet the predefined threshold for entry into the multivariable model and were therefore not retained.”*

**5. Interpretation of Non-Significant Results
Some variables (e.g., breastfeeding, smartphone use) are discussed as trends despite non-significance in multivariate analysis.
• Avoid overinterpretation of non-significant findings.
• Clearly distinguish statistically significant vs. non-significant associations.**

**Response:** We thank the reviewer for this important comment. We agree that non-significant findings should be interpreted with caution and clearly distinguished from statistically significant associations. In the revised manuscript, we have therefore refined both the Results and Discussion sections to make this distinction more explicit.

Specifically, we now state clearly which variables remained significantly associated with myopia in the multivariable model (parental history, school cycle, urban school location, and physical activity) and which variables were included in the model but did not remain statistically significant after adjustment (such as breastfeeding duration and smartphone use). We also revised the Discussion to avoid presenting these non-significant variables as trends with substantive interpretative weight. Instead, they are now described more cautiously as non-significant findings that may reflect residual confounding, limited precision, or overlap with other variables retained in the model.

These revisions have been incorporated into the Results and Discussion sections.

Results:

*“Other variables included in the model, such as breastfeeding duration and smartphone use (during the week and on weekends), did not remain statistically significant after adjustment (p > 0.05).”*

Discussion:

*“Regarding behavioural factors, regular physical activity (≥ 2 times/week) was associated with lower rates of myopia (OR = 0.471), corresponding to an approximate 53% reduction in odds. Although the literature often highlights time spent outdoors and smartphone use as relevant correlates of myopia [8,11,27,29], in our data outdoor activity did not meet the predefined threshold for inclusion in the multivariable model, whereas smartphone use was included but did not maintain statistical significance after adjustment. This lack of significance, also observed in similar studies [16,28], may be attributed to the "complete-case" approach and the control of confounding effects. For example, the associated observed for digital devices use may be partially explain by the higher academic demands of the 3rd cycle or attenuated by the strong influence of parental history in this specific sample. Similarly, breastfeeding duration was retained in the model but did not show an independent association after adjustment. These findings should therefore be interpreted cautiously and not as evidence of independent effects in this sample.”*

**6. Potential Confounding Factors Not Considered
Important variables were not included or directly measured:
• Axial length
• Objective outdoor light exposure
• Socioeconomic status (beyond parental education)
Amended
• Expand discussion on residual confounding.
• Justify omission of key variables.**

**Response:** We thank the reviewer for this important comment and agree with the observation. We have expanded the Limitations section to acknowledge the possibility of residual confounding due to variables not directly measured in this study. Specifically, axial length was not assessed because the protocol was designed for large-scale school-based screening, which limited the feasibility of using higher-precision equipment such as ocular biometry. We also clarify that outdoor exposure was assessed through self-reported behavioural measures rather than objective light exposure monitoring and therefore may not fully capture the intensity or duration of ambient light exposure. In addition, socioeconomic status was only partially represented by parental education level; broader socioeconomic indicators, such as household income, occupation, or material deprivation, were not available.

We now explicitly acknowledge that these unmeasured or incompletely measured factors may have contributed to residual confounding and may have influenced the magnitude of some observed associations. These clarifications have been incorporated into the Discussion/Limitations section.

Discussion (limitations):

*“5) Finally, residual confounding cannot be excluded, as some relevant variables were not directly measured in this study. Axial length, an important structural correlate of myopia, was not assessed because the screening protocol was designed for large-scale school-based refractive evaluation and did not support the use of higher-precision equipment such as ocular biometry. Likewise, outdoor exposure was assessed through self-reported time outdoors rather than objective light exposure measurements, and therefore may not fully capture actual ambient light exposure. Socioeconomic status was only partially represented by parental education level, while other potentially relevant indicators, such as household income, occupation, or material deprivation, were not available. These factors may have contributed to residual confounding and should be considered in the interpretation of the observed associations.”*

**7. Data Availability Statement (Compliance Issue)
The manuscript states that data are available “upon request,” which does not meet PLOS ONE data policy.
• Deposit data in a public repository (e.g., Dryad, Figshare).
• Provide DOI or accession number.
• Revise statement accordingly.**

**Response:**
We thank the reviewer for this important comment. In accordance with PLOS ONE data-sharing requirements, the anonymized minimal dataset underlying the findings of this study has been deposited in the Zenodo repository and assigned the following DOI: <https://doi.org/10.5281/zenodo.19423653>. The Data Availability Statement has been revised accordingly in both the submission system and the manuscript.

**Data Availability Statement**
The anonymized minimal dataset underlying the findings of this study is publicly available in Zenodo at <https://doi.org/10.5281/zenodo.19423653>

**8. Sample Representativeness
The study is limited to one region with low population density.
• Discuss generalizability limitations more explicitly.
• Clarify whether the sample is representative of Portuguese adolescents.**

**Response:**
We thank the reviewer for this important comment. We agree that the sample was drawn from a specific school-based population in a low-population-density region of central Portugal and should not be interpreted as nationally representative of all Portuguese adolescents. In the revised manuscript, we now state this more explicitly and clarify that the findings are most applicable to adolescents from similar non-metropolitan school settings. We also emphasize that caution is needed when extrapolating these results to the broader Portuguese adolescent population, particularly to more urbanized or socioeconomically different contexts. These clarifications have been incorporated into the Discussion/Limitations section.

Limitation

*“2) The final analytic sample (N = 651) was reduced by the complete-case approach*, *largely because of missing data in the parental questionnaire, particularly for family history of myopia. As these missing data may not have been completely random, some degree of selection bias cannot be excluded, and the magnitude of certain adjusted associations may have been affected. In addition, because participants were recruited through schools, in a single region of central Portugal, the sample should not be considered nationally representative. Although school setting/location was included as an adjustment variable, the limited number of schools precluded a more robust multilevel approach. While this may affect generalizability, the model maintained an acceptable Events Per Variable (EPV) ratio, supporting its statistical stability within this specific regional context. Therefore, the findings are most applicable to adolescents from similar non-metropolitan school settings and should be extrapolated cautiously to broad Portuguese adolescent population.”*

**1. Language and Grammar
Several minor issues:
• “portuguese adolescents” → should be capitalized
• “sit in the front row” → “sitting in the front row”
• “Tittle” → “Title”
Please full professional language editing.**

**Response:**
We thank the reviewer for this comment. We have carefully revised the manuscript for language, grammar, spelling, capitalization, and terminology consistency throughout. The specific issues highlighted by the reviewer were corrected, and additional editing was performed to improve the overall clarity and fluency of the text in standard scientific English.

**2. Clarity of Methods
• Clarify definition of “urban vs. rural” schools
• Provide questionnaire validation details
• Specify how screen time categories were derived**

**Response:**
We thank the reviewer for this important comment. We have clarified the Methods section in three ways. First, we now specify the operational definition of school location based on the Portuguese National Statistics Institute (INE) criteria, explicitly distinguishing schools located in Predominantly Urban Areas from those located in Moderately Urban Areas within the regional studied context. Second, we expanded the description of questionnaire development by clarifying that the instrument was literature-based, reviewed by a multidisciplinary panel, pilot-tested, and administered under supervised classroom conditions; we also clarify that it was a study-specific instrument rather than a previously standardized questionnaire. Third, we now specify how screen time categories were derived, indicating that smartphone use was self-reported separately for weekdays and weekends and categorized into predefined time intervals. These clarifications have been incorporated into the Methods section.

*“The potential factors correlated with myopia, were collected through a questionnaire. The instrument was developed based on an extensive literature review and refined by a multidisciplinary committee comprising eye care professionals, a physician, and a behavioural psychologist to ensure clinical relevance and minimize reporting bias. To assess clarity and cultural appropriateness, a pilot study was conducted with a representative class (data not included in the final analysis), leading to minor adjustments in wording. Although the questionnaire was not a previously validated standardized instrument, these steps were taken to enhance content relevance and comprehension. During the main data collection, the instruments were administered in a supervised classroom environment to minimize peer influence and social desirability bias. Information on perinatal and postnatal characteristics, as well as parental myopia history, was provided by the students' parents, via printed forms distributed and collected by the class directors. Demographic, educational, and behavioural data were self-reported by the students. With the exception of age, questionnaire-derived variables were collected using predefined categorical response options. All response categories used in the analyses are presented in Table 2. (…)*

*School locations were classified according to the Portuguese National Statistics Institute (INE) criteria. Schools located in Predominantly Urban Areas (APU) were classified as Urban, whereas schools located in Moderately Urban Areas (AMU) were classified as Rural/Semi-urban for analytical contrast within this low-population-density regional context. No schools from Predominantly Rural Areas (APR) were included in the study.”*

**3. Statistical Reporting
• Use consistent decimal format (e.g., 0.05 vs 0,05)
• Report exact p-values where possible
• Ensure all ORs include confidence intervals**

**Response:**
We thank the reviewer for this comment. We carefully revised the statistical reporting throughout the manuscript. Decimal notation was standardized using decimal points throughout the text and tables, exact p-values were reported wherever appropriate, and all odds ratios were checked to ensure that corresponding 95% confidence intervals were provided. These revisions were incorporated into the Abstract, Results, and Tables 2 and 3.

**4. Tables Presentation
• Table 2 is dense and difficult to read
• Consider splitting into:
o Demographic factors
o Behavioral factors
• Highlight significant results more clearly**

**Response:**
We thank the reviewer for this helpful suggestion. We agree that the original Table 2 was difficult to read in its previous format. In the revised manuscript, we have split it into Table 2A (demographic, educational, perinatal/postnatal, and familial factors) and Table 2B (behavioural and environmental factors). In addition, statistically significant findings remain highlighted in bold, and the revised table layout improves their readability.

**5. Discussion Section
• Some repetition of results (especially OR values)
• Could benefit from deeper comparison with recent European studies (2020–2025)**

**Response:**
We thank the reviewer for this helpful comment. We revised the Discussion to reduce repetition of results already presented in the tables, particularly odds ratios, and to strengthen comparison with recent European studies. These changes have now been incorporated into the manuscript.

**6. Ethics Statement
Ethics approval is provided, but:
• Consent procedures (written vs verbal) should be clarified more explicitly in Methods.**

**Response:**

We now state that written informed consent was obtained from the parents or legal guardians of all participating minors, and that verbal assent was obtained from the adolescents on the day of assessment.

**7. Terminology Consistency
• Use consistent terms:
o “smartphone use” vs “digital device use”
o “outdoor time” vs “outdoor activities”**

**Response:**
We thank the reviewer for this helpful comment. We have revised the manuscript to ensure greater terminology consistency throughout. Specifically, we now use **“smartphone use”** when referring to the measured exposure variable and **“outdoor time”** when referring to time spent outdoors, including weekday and weekend exposure. These terms have been standardized throughout the text and tables.

**Reviewer #5:** **The abstract is well written, clear, and logically structured. It effectively outlines the study’s purpose, methodology, key results, and conclusions in a concise and coherent manner. The objectives are clearly defined, the methods are appropriately described, and the main findings are presented with sufficient detail to support the conclusions. The language is precise and accessible, allowing the reader to بسهولة grasp the relevance and implications of the study. Overall, the abstract provides a comprehensive and informative summary of the work, and it is suitable for publication in its current form without the need for revisions.**

**Response:**
We thank the reviewer for this positive and encouraging assessment of the abstract. We appreciate the recognition of its clarity, structure, and overall suitability for publication.
