# Supplementary material for: Associated factors of myopia in a Portuguese sample of adolescents: Parental history and school-related lifestyle
Source: PLoS One. 2026 Jul 16;21(7):e0353283. doi: 10.1371/journal.pone.0353283 (PMC13375035; doi:10.1371/journal.pone.0353283)
Supplement: Peer review history

# Peer review: Decision Letter 0

**DOI:** 10.1371/journal.pone.0353283.r001

**Authors:** Andrzej Grzybowski

4 Mar 2026

PONE-D-26-00450

Prevalence of Myopia and Associated Factors in Adolescents in a Central Region of Portugal

PLOS One

Dear Dr. Nunes,

Thank you for submitting your manuscript to PLOS ONE. After careful consideration, we feel that it has merit but does not fully meet PLOS ONE’s publication criteria as it currently stands. Therefore, we invite you to submit a revised version of the manuscript that addresses the points raised during the review process.

Please submit your revised manuscript by Apr 18 2026 11:59PM. If you will need more time than this to complete your revisions, please reply to this message or contact the journal office at plosone@plos.org. When you're ready to submit your revision, log on to https://www.editorialmanager.com/pone/ and select the ’submissions Needing Revision' folder to locate your manuscript file.

Please include the following items when submitting your revised manuscript:

- A letter that responds to each point raised by the academic editor and reviewer(s). You should upload this letter as a separate file labeled 'Response to Reviewers'.
- A marked-up copy of your manuscript that highlights changes made to the original version. You should upload this as a separate file labeled 'Revised Manuscript with Track Changes'.
- An unmarked version of your revised paper without tracked changes. You should upload this as a separate file labeled 'Manuscript'.

If you would like to make changes to your financial disclosure, please include your updated statement in your cover letter. Guidelines for resubmitting your figure files are available below the reviewer comments at the end of this letter.

If applicable, we recommend that you deposit your laboratory protocols in protocols.io to enhance the reproducibility of your results. Protocols.io assigns your protocol its own identifier (DOI) so that it can be cited independently in the future. For instructions see: https://journals.plos.org/plosone/s/submission-guidelines#loc-laboratory-protocols. Additionally, PLOS ONE offers an option for publishing peer-reviewed Lab Protocol articles, which describe protocols hosted on protocols.io. Read more information on sharing protocols at https://plos.org/protocols?utm_medium=editorial-email&utm_source=authorletters&utm_campaign=protocols.

We look forward to receiving your revised manuscript.

Kind regards,

Andrzej Grzybowski

Academic Editor

PLOS One

Journal Requirements:

When submitting your revision, we need you to address these additional requirements.

1. Please ensure that your manuscript meets PLOS ONE’s style requirements, including those for file naming. The PLOS ONE style templates can be found at

https://journals.plos.org/plosone/s/file?id=wjVg/PLOSOne_formatting_sample_main_body.pdf and https://journals.plos.org/plosone/s/file?id=ba62/PLOSOne_formatting_sample_title_authors_affiliations.pdf

2. Please provide additional details regarding participant consent. In the ethics statement in the Methods and online submission information, please ensure that you have specified (1) whether consent was informed and (2) what type you obtained (for instance, written or verbal, and if verbal, how it was documented and witnessed). If your study included minors, state whether you obtained consent from parents or guardians. If the need for consent was waived by the ethics committee, please include this information.

3. In the online submission form you indicate that your data is not available for proprietary reasons and have provided a contact point for accessing this data. Please note that your current contact point is a co-author on this manuscript. According to our Data Policy, the contact point must not be an author on the manuscript and must be an institutional contact, ideally not an individual. Please revise your data statement to a non-author institutional point of contact, such as a data access or ethics committee, and send this to us via return email. Please also include contact information for the third-party organization, and please include the full citation of where the data can be found.

4. Please include your full ethics statement in the “Methods” section of your manuscript file. In your statement, please include the full name of the IRB or ethics committee who approved or waived your study, as well as whether or not you obtained informed written or verbal consent. If consent was waived for your study, please include this information in your statement as well.

If the reviewer comments include a recommendation to cite specific previously published works, please review and evaluate these publications to determine whether they are relevant and should be cited. There is no requirement to cite these works unless the editor has indicated otherwise.

[Note: HTML markup is below. Please do not edit.]

Reviewers' comments:

Reviewer’s Responses to Questions

**Comments to the Author**

1. Is the manuscript technically sound, and do the data support the conclusions?

The manuscript must describe a technically sound piece of scientific research with data that supports the conclusions. Experiments must have been conducted rigorously, with appropriate controls, replication, and sample sizes. The conclusions must be drawn appropriately based on the data presented.

Reviewer #1: Partly

Reviewer #2: Yes

Reviewer #3: Yes

**********

2. Has the statistical analysis been performed appropriately and rigorously?

Reviewer #1: No

Reviewer #2: Yes

Reviewer #3: Yes

**********

3. Have the authors made all data underlying the findings in their manuscript fully available?

The PLOS Data policy requires authors to make all data underlying the findings described in their manuscript fully available without restriction, with rare exception (please refer to the Data Availability Statement in the manuscript PDF file). The data should be provided as part of the manuscript or its supporting information, or deposited to a public repository. For example, in addition to summary statistics, the data points behind means, medians and variance measures should be available. If there are restrictions on publicly sharing data—e.g. participant privacy or use of data from a third party—those must be specified.

Reviewer #1: Yes

Reviewer #2: Yes

Reviewer #3: No

**********

4. Is the manuscript presented in an intelligible fashion and written in standard English?

PLOS ONE does not copyedit accepted manuscripts, so the language in submitted articles must be clear, correct, and unambiguous. Any typographical or grammatical errors should be corrected at revision, so please note any specific errors here.

Reviewer #1: Yes

Reviewer #2: Yes

Reviewer #3: Yes

**********

5. Review Comments to the Author

Please use the space provided to explain your answers to the questions above. You may also include additional comments for the author, including concerns about dual publication, research ethics, or publication ethics. (Please upload your review as an attachment if it exceeds 20,000 characters)

Reviewer #1: 1. The introduction is weak and would benefit from a clearer rationale and concise review of relevant literature.

2. Discussion of environmental factors should reference the recent review: DOI: 10.1186/s40101-024-00354-7.

3. The use of non-cycloplegic autorefraction in children (Lines 99–100) is a limitation, as Plusoptix may not be reliable across all refractive errors (Ref #19, OPO 2025).

4. Sample size is low, potentially limiting statistical power and generalizability.

5. Self-reported data from parents and children may be biased (especially lifestyle questions); the manuscript should clarify whether any validation or cross-checking was implemented.

6. The criteria for classifying locations as protective against myopia or as urban/rural are unclear.

7. Numerical formatting is inconsistent; please standardize (preferably using decimals).

8. The rationale for selecting the 10–18 year age range is not provided.

9. Statistical analysis does not account for correlation between fellow eyes when using both eyes in logistic regression; appropriate methods should be applied.

10. Overall: The study addresses an important topic, but methodological limitations and unclear reporting reduce the reliability and interpretability of the findings. Revision addressing these issues is recommended.

Reviewer #2: Dear Authors,

Thank you for the submission. The dataset is clearly valuable; however, because the underlying screening campaign has already been published, this manuscript must clearly differentiate itself and add substantial new value.

1) Make the novelty explicit (required).

Please state explicitly (Abstract + Methods) that this is a secondary analysis/subsample of the previously published dataset, provide a participant flow (original screened N → analytic N), and add a short “What is new vs. prior publication” paragraph/table. Manuscripts based on the same database must substantially add and be transparent about overlap.

2) Shift the focus: this should be a “school system / digital exposure” paper, not another prevalence paper.

De-emphasize prevalence as a headline result and reframe around:

differences between school cycles/grades and academic demands,

weekend vs weekday digital device patterns,

urban vs rural school context and how it structures behavior (outdoor time/physical activity),

with clear, school-relevant implications (screening and prevention in schools). This is where the paper can genuinely stand out.

3) Screen-time interpretation: be precise.

You measure mainly smartphone/digital device time. Current evidence suggests device type matters and smartphone associations are not always the strongest signal compared with computers/TV; interpret as a proxy of indoor/digital lifestyle and discuss this limitation explicitly.

4) Fix technical credibility issues (before any interpretation).

Correct the implausible/inconsistent counts in the tables and ensure p-values/OR/CI are internally consistent. Then strengthen modelling (avoid/justify stepwise selection; account for school clustering; sensitivity analysis given non‑cycloplegic refraction threshold)

Reviewer #3: a) Major Points

1 Cycloplegia / refractive assessment: Refractive error was measured without cycloplegia. Although the use of an open-field autorefractor may reduce accommodative effects, the absence of cycloplegia remains an important limitation in this age group and should be clearly acknowledged due to the potential overestimation of myopia prevalence.

2 Interpretation of prevalence: The reported prevalence of 22.5% falls within the range described in other European studies. Statements describing this prevalence as “significant” or implying a strong public health burden should be moderated.

3 Interpretation of associated factors: Some associations should be interpreted with greater caution. For example, the association with classroom seating position may reflect reverse causation (students with myopia may preferentially sit closer to the board) rather than a true risk factor.

4 Multivariate model interpretation: The reporting of the multivariate model could be clarified. Variables such as breastfeeding and smartphone use appear in the final model despite not reaching statistical significance. The manuscript should clearly distinguish between variables retained in the model and those with statistically significant associations.

5 Length of the discussion and conclusion: The discussion is relatively long and could be streamlined. Several sections repeat results already presented in the tables. The conclusion could also be shortened and focus more directly on the main findings rather than broader public health implications that are not directly supported by the study design.

b) Minor / Specific Points

1 Abstract

• The conclusion is somewhat long and could be shortened to better reflect the key findings.

• The absence of cycloplegic refraction should be mentioned as an important limitation.

• Associations such as classroom seating position should be interpreted cautiously.

2 Introduction

• The introduction could be streamlined to improve clarity and avoid overly strong statements.

• For example, describing myopia as the “leading cause of correctable vision loss among young people” seems overly strong; wording such as vision impairment would be more appropriate.

• The specific knowledge gap addressed by the study should be stated more clearly.

• Please clarify the rationale for focusing specifically on adolescents aged 10–18 years.

• The phrase “estimate the proportion of adolescents with myopia” could be replaced with “estimate the prevalence of myopia.”

3 Methods

• The contextual description of the study region (e.g., altitude, population density) could be shortened, as these variables are not directly analyzed in the study.

4 Results

• Parts of the narrative repeat information already shown in the tables (particularly Table 2). The text could focus more on the key findings.

• Some wording could be simplified (e.g., replace “greater probability of myopia” with “higher odds of myopia”).

• The terminology used for breastfeeding categories should be revised. Expressions such as “Didn’t do it” are not appropriate for a scientific manuscript and could be replaced with “No breastfeeding.”

5 Discussion

• Some interpretations appear slightly overstated and could be moderated.

• Several paragraphs could be shortened, particularly where findings are repeatedly compared with the literature.

**********

6. PLOS authors have the option to publish the peer review history of their article (what does this mean?). If published, this will include your full peer review and any attached files.

If you choose “no”, your identity will remain anonymous but your review may still be made public.

**Do you want your identity to be public for this peer review?**  For information about this choice, including consent withdrawal, please see our Privacy Policy.

Reviewer #1: No

Reviewer #2: No

Reviewer #3: No

**********

[NOTE: If reviewer comments were submitted as an attachment file, they will be attached to this email and accessible via the submission site. Please log into your account, locate the manuscript record, and check for the action link "View Attachments". If this link does not appear, there are no attachment files.]

To ensure your figures meet our technical requirements, please review our figure guidelines: https://journals.plos.org/plosone/s/figures

You may also use PLOS’s free figure tool, NAAS, to help you prepare publication quality figures: https://journals.plos.org/plosone/s/figures#loc-tools-for-figure-preparation.

NAAS will assess whether your figures meet our technical requirements by comparing each figure against our figure specifications.

# Author comment: Author response to Decision Letter 1

**DOI:** 10.1371/journal.pone.0353283.r002

17 Apr 2026

Point-by-point responses to the Editor and reviewers follow in the attached document, entitled "ReplyReview

Attachment Submitted filename: ReplyReviewersvf.docx

# Peer review: Decision Letter 1

**DOI:** 10.1371/journal.pone.0353283.r003

**Authors:** Andrzej Grzybowski, Andrzej Grzybowski

19 May 2026

PONE-D-26-00450R1

Associated factors of myopia in a Portuguese sample of adolescents: Parental history and school-related lifestyle

PLOS One

Dear Dr. Nunes,

Thank you for submitting your manuscript to PLOS ONE. After careful consideration, we feel that it has merit but does not fully meet PLOS ONE’s publication criteria as it currently stands. Therefore, we invite you to submit a revised version of the manuscript that addresses the points raised during the review process.

Please submit your revised manuscript by Jul 03 2026 11:59PM. If you will need more time than this to complete your revisions, please reply to this message or contact the journal office at plosone@plos.org. When you're ready to submit your revision, log on to https://www.editorialmanager.com/pone/ and select the ’submissions Needing Revision' folder to locate your manuscript file.

Please include the following items when submitting your revised manuscript:

- A letter that responds to each point raised by the academic editor and reviewer(s). You should upload this letter as a separate file labeled 'Response to Reviewers'.
- A marked-up copy of your manuscript that highlights changes made to the original version. You should upload this as a separate file labeled 'Revised Manuscript with Track Changes'.
- An unmarked version of your revised paper without tracked changes. You should upload this as a separate file labeled 'Manuscript'.

If you would like to make changes to your financial disclosure, please include your updated statement in your cover letter. Guidelines for resubmitting your figure files are available below the reviewer comments at the end of this letter.

If applicable, we recommend that you deposit your laboratory protocols in protocols.io to enhance the reproducibility of your results. Protocols.io assigns your protocol its own identifier (DOI) so that it can be cited independently in the future. For instructions see: https://journals.plos.org/plosone/s/submission-guidelines#loc-laboratory-protocols. Additionally, PLOS ONE offers an option for publishing peer-reviewed Lab Protocol articles, which describe protocols hosted on protocols.io. Read more information on sharing protocols at https://plos.org/protocols?utm_medium=editorial-email&utm_source=authorletters&utm_campaign=protocols.

As the corresponding author, your ORCID iD is verified in the submission system and will appear in the published article. PLOS supports the use of ORCID, and we encourage all coauthors to register for an ORCID iD and use it as well. Please encourage your coauthors to verify their ORCID iD within the submission system before final acceptance, as unverified ORCID iDs will not appear in the published article. *Only*  the individual author can complete the verification step; PLOS staff *cannot*  verify ORCID iDs on behalf of authors.

We look forward to receiving your revised manuscript.

Kind regards,

Andrzej Grzybowski

Academic Editor

PLOS One

**Journal Requirements:**

1. If the reviewer comments include a recommendation to cite specific previously published works, please review and evaluate these publications to determine whether they are relevant and should be cited. There is no requirement to cite these works unless the editor has indicated otherwise.

2. Please review your reference list to ensure that it is complete and correct. If you have cited papers that have been retracted, please include the rationale for doing so in the manuscript text, or remove these references and replace them with relevant current references. Any changes to the reference list should be mentioned in the rebuttal letter that accompanies your revised manuscript. If you need to cite a retracted article, indicate the article’s retracted status in the References list and also include a citation and full reference for the retraction notice.

[Note: HTML markup is below. Please do not edit.]

Reviewers' comments:

Reviewer’s Responses to Questions

**Comments to the Author**

1. If the authors have adequately addressed your comments raised in a previous round of review and you feel that this manuscript is now acceptable for publication, you may indicate that here to bypass the “Comments to the Author” section, enter your conflict of interest statement in the “Confidential to Editor” section, and submit your "Accept" recommendation.

Reviewer #1: All comments have been addressed

Reviewer #4: (No Response)

Reviewer #5: (No Response)

**********

2. Is the manuscript technically sound, and do the data support the conclusions?

The manuscript must describe a technically sound piece of scientific research with data that supports the conclusions. Experiments must have been conducted rigorously, with appropriate controls, replication, and sample sizes. The conclusions must be drawn appropriately based on the data presented.

Reviewer #1: Yes

Reviewer #4: Yes

Reviewer #5: Yes

**********

3. Has the statistical analysis been performed appropriately and rigorously?

Reviewer #1: Yes

Reviewer #4: I Don't Know

Reviewer #5: Yes

**********

4. Have the authors made all data underlying the findings in their manuscript fully available?

The PLOS Data policy requires authors to make all data underlying the findings described in their manuscript fully available without restriction, with rare exception (please refer to the Data Availability Statement in the manuscript PDF file). The data should be provided as part of the manuscript or its supporting information, or deposited to a public repository. For example, in addition to summary statistics, the data points behind means, medians and variance measures should be available. If there are restrictions on publicly sharing data—e.g. participant privacy or use of data from a third party—those must be specified.

Reviewer #1: Yes

Reviewer #4: Yes

Reviewer #5: Yes

**********

5. Is the manuscript presented in an intelligible fashion and written in standard English?

PLOS ONE does not copyedit accepted manuscripts, so the language in submitted articles must be clear, correct, and unambiguous. Any typographical or grammatical errors should be corrected at revision, so please note any specific errors here.

Reviewer #1: Yes

Reviewer #4: No

Reviewer #5: Yes

**********

6. Review Comments to the Author

Please use the space provided to explain your answers to the questions above. You may also include additional comments for the author, including concerns about dual publication, research ethics, or publication ethics. (Please upload your review as an attachment if it exceeds 20,000 characters)

**Reviewer #1:**  (No Response)

**Reviewer #4:**  Thank you for your invitation to review manuscript entitled" Title: Prevalence of Myopia and Associated Factors in Adolescents in a Central Region of Portugal

1. Use of Non-Cycloplegic Refraction (Key Methodological Limitation)

The study measures myopia without cycloplegia, which is known to overestimate myopia prevalence, particularly in children and adolescents due to accommodation bias.

• Strengthen justification for this choice in the Methods.

• Expand discussion on potential overestimation magnitude.

• Clearly state how this limitation affects comparability with other studies.

2. Cross-Sectional Design and Causality

The cross-sectional design limits causal inference, yet some interpretations suggest directional relationships (e.g., smartphone use → myopia).

• Rephrase conclusions to emphasize association, not causation.

• Avoid causal language such as “increases risk” or “protective effect” without caution.

3. Handling of Missing Data

Approximately 20% missing data for parental myopia is substantial.

• Explain how missing data were handled (e.g., exclusion, imputation).

• Include sensitivity analysis if possible.

• Discuss potential bias introduced by missing parental data.

4. Variable Selection in Multivariate Model

The manuscript does not clearly describe:

• Criteria for including variables in the multivariate model

• Whether collinearity was assessed

Amended

• Specify selection criteria (e.g., p < 0.20 in univariate analysis).

• Report multicollinearity diagnostics (e.g., VIF).

• Clarify why some variables (e.g., age, outdoor time) lost significance.

5. Interpretation of Non-Significant Results

Some variables (e.g., breastfeeding, smartphone use) are discussed as trends despite non-significance in multivariate analysis.

• Avoid overinterpretation of non-significant findings.

• Clearly distinguish statistically significant vs. non-significant associations.

6. Potential Confounding Factors Not Considered

Important variables were not included or directly measured:

• Axial length

• Objective outdoor light exposure

• Socioeconomic status (beyond parental education)

Amended

• Expand discussion on residual confounding.

• Justify omission of key variables.

7. Data Availability Statement (Compliance Issue)

The manuscript states that data are available “upon request,” which does not meet PLOS ONE data policy.

• Deposit data in a public repository (e.g., Dryad, Figshare).

• Provide DOI or accession number.

• Revise statement accordingly.

8. Sample Representativeness

The study is limited to one region with low population density.

• Discuss generalizability limitations more explicitly.

• Clarify whether the sample is representative of Portuguese adolescents.

1. Language and Grammar

Several minor issues:

• “portuguese adolescents” → should be capitalized

• “sit in the front row” → “sitting in the front row”

• “Tittle” → “Title”

Please full professional language editing.

2. Clarity of Methods

• Clarify definition of “urban vs. rural” schools

• Provide questionnaire validation details

• Specify how screen time categories were derived

3. Statistical Reporting

• Use consistent decimal format (e.g., 0.05 vs 0,05)

• Report exact p-values where possible

• Ensure all ORs include confidence intervals

4. Tables Presentation

• Table 2 is dense and difficult to read

• Consider splitting into:

o Demographic factors

o Behavioral factors

• Highlight significant results more clearly

5. Discussion Section

• Some repetition of results (especially OR values)

• Could benefit from deeper comparison with recent European studies (2020–2025)

6. Ethics Statement

Ethics approval is provided, but:

• Consent procedures (written vs verbal) should be clarified more explicitly in Methods.

7. Terminology Consistency

• Use consistent terms:

o “smartphone use” vs “digital device use”

o “outdoor time” vs “outdoor activities”

**Reviewer #5:**  The abstract is well written, clear, and logically structured. It effectively outlines the study’s purpose, methodology, key results, and conclusions in a concise and coherent manner. The objectives are clearly defined, the methods are appropriately described, and the main findings are presented with sufficient detail to support the conclusions. The language is precise and accessible, allowing the reader to بسهولة grasp the relevance and implications of the study. Overall, the abstract provides a comprehensive and informative summary of the work, and it is suitable for publication in its current form without the need for revisions.

**********

7. PLOS authors have the option to publish the peer review history of their article (what does this mean?). If published, this will include your full peer review and any attached files.

If you choose “no”, your identity will remain anonymous but your review may still be made public.

**Do you want your identity to be public for this peer review?**  For information about this choice, including consent withdrawal, please see our Privacy Policy.

Reviewer #1: No

Reviewer #4: No

Reviewer #5: No

**********

[NOTE: If reviewer comments were submitted as an attachment file, they will be attached to this email and accessible via the submission site. Please log into your account, locate the manuscript record, and check for the action link "View Attachments". If this link does not appear, there are no attachment files.]

To ensure your figures meet our technical requirements, please review our figure guidelines: https://journals.plos.org/plosone/s/figures

You may also use PLOS’s free figure tool, NAAS, to help you prepare publication quality figures: https://journals.plos.org/plosone/s/figures#loc-tools-for-figure-preparation.

NAAS will assess whether your figures meet our technical requirements by comparing each figure against our figure specifications.

# Author comment: Author response to Decision Letter 2

**DOI:** 10.1371/journal.pone.0353283.r004

16 Jun 2026

A point-by-point response to the reviewers' comments was attached to the reviewers' response document.

Attachment Submitted filename: Response to Reviewers_2nd.doc

# Peer review: Decision Letter 2

**DOI:** 10.1371/journal.pone.0353283.r005

**Authors:** Andrzej Grzybowski, Andrzej Grzybowski, Andrzej Grzybowski

22 Jun 2026

Associated factors of myopia in a Portuguese sample of adolescents: Parental history and school-related lifestyle

PONE-D-26-00450R2

Dear Dr. Nunes,

We’re pleased to inform you that your manuscript has been judged scientifically suitable for publication and will be formally accepted for publication once it meets all outstanding technical requirements.

Within one week, you’ll receive an e-mail detailing the required amendments. When these have been addressed, you’ll receive a formal acceptance letter and your manuscript will be scheduled for publication.

An invoice will be generated when your article is formally accepted. Please note, if your institution has a publishing partnership with PLOS and your article meets the relevant criteria, all or part of your publication costs will be covered. Please make sure your user information is up-to-date by logging into Editorial Manager at Editorial Manager® and clicking the ‘Update My Information' link at the top of the page. For questions related to billing, please contact billing support.

If your institution or institutions have a press office, please notify them about your upcoming paper to help maximize its impact. If they’ll be preparing press materials, please inform our press team as soon as possible -- no later than 48 hours after receiving the formal acceptance. Your manuscript will remain under strict press embargo until 2 pm Eastern Time on the date of publication. For more information, please contact onepress@plos.org.

Kind regards,

Andrzej Grzybowski

Academic Editor

PLOS One

Additional Editor Comments (optional):

Reviewers' comments:

Reviewer’s Responses to Questions

**Comments to the Author**

1. If the authors have adequately addressed your comments raised in a previous round of review and you feel that this manuscript is now acceptable for publication, you may indicate that here to bypass the “Comments to the Author” section, enter your conflict of interest statement in the “Confidential to Editor” section, and submit your "Accept" recommendation.

Reviewer #4: All comments have been addressed

**********

2. Is the manuscript technically sound, and do the data support the conclusions?

The manuscript must describe a technically sound piece of scientific research with data that supports the conclusions. Experiments must have been conducted rigorously, with appropriate controls, replication, and sample sizes. The conclusions must be drawn appropriately based on the data presented.

Reviewer #4: Yes

**********

3. Has the statistical analysis been performed appropriately and rigorously?

Reviewer #4: Yes

**********

4. Have the authors made all data underlying the findings in their manuscript fully available?

The PLOS Data policy requires authors to make all data underlying the findings described in their manuscript fully available without restriction, with rare exception (please refer to the Data Availability Statement in the manuscript PDF file). The data should be provided as part of the manuscript or its supporting information, or deposited to a public repository. For example, in addition to summary statistics, the data points behind means, medians and variance measures should be available. If there are restrictions on publicly sharing data—e.g. participant privacy or use of data from a third party—those must be specified.

Reviewer #4: Yes

**********

5. Is the manuscript presented in an intelligible fashion and written in standard English?

PLOS ONE does not copyedit accepted manuscripts, so the language in submitted articles must be clear, correct, and unambiguous. Any typographical or grammatical errors should be corrected at revision, so please note any specific errors here.

Reviewer #4: Yes

**********

6. Review Comments to the Author

Please use the space provided to explain your answers to the questions above. You may also include additional comments for the author, including concerns about dual publication, research ethics, or publication ethics. (Please upload your review as an attachment if it exceeds 20,000 characters)

Reviewer #4: The manuscript has been amended thoroughly and appropriately. Based on a thorough review of the revised manuscript and the detailed point-by-point response provided by the authors, it is clear that the authors have made a significant and commendable effort to address the vast majority of the reviewer’s comments.

**********

7. PLOS authors have the option to publish the peer review history of their article (what does this mean?). If published, this will include your full peer review and any attached files.

If you choose “no”, your identity will remain anonymous but your review may still be made public.

**Do you want your identity to be public for this peer review?**  For information about this choice, including consent withdrawal, please see our Privacy Policy.

Reviewer #4: No

**********

# Editor report: Acceptance letter

**DOI:** 10.1371/journal.pone.0353283.r006

**Authors:** Andrzej Grzybowski, Andrzej Grzybowski, Andrzej Grzybowski

PONE-D-26-00450R2

PLOS One

Dear Dr. Nunes,

I'm pleased to inform you that your manuscript has been deemed suitable for publication in PLOS One. Congratulations! Your manuscript is now being handed over to our production team.

At this stage, our production department will prepare your paper for publication. This includes ensuring the following:

* All references, tables, and figures are properly cited

* All relevant supporting information is included in the manuscript submission,

* There are no issues that prevent the paper from being properly typeset

You will receive further instructions from the production team, including instructions on how to review your proof when it is ready. Please keep in mind that we are working through a large volume of accepted articles, so please give us a few days to review your paper and let you know the next and final steps.

Lastly, if your institution or institutions have a press office, please let them know about your upcoming paper now to help maximize its impact. If they'll be preparing press materials, please inform our press team within the next 48 hours. Your manuscript will remain under strict press embargo until 2 pm Eastern Time on the date of publication. For more information, please contact onepress@plos.org.

You will receive an invoice from PLOS for your publication fee after your manuscript has reached the completed accept phase. If you receive an email requesting payment before acceptance or for any other service, this may be a phishing scheme. Learn how to identify phishing emails and protect your accounts at https://explore.plos.org/phishing.

If we can help with anything else, please email us at customercare@plos.org.

Thank you for submitting your work to PLOS ONE and supporting open access.

Kind regards,

PLOS ONE Editorial Office Staff

on behalf of

Dr. Andrzej Grzybowski

Academic Editor

PLOS One
